# On the proposed structures and stereocontrolled synthesis of the cephalosporolides

**DOI:** 10.3762/bjoc.8.146

**Published:** 2012-08-14

**Authors:** Sami F Tlais, Gregory B Dudley

**Affiliations:** 1 Department of Chemistry and Biochemistry, Florida State University, Tallahassee, FL 32306-4390 USA, Fax: (850) 644-8281

**Keywords:** cephalosporolides, chelation, spiroketals, stereocontrol, zinc chloride

## Abstract

The synthesis of four candidate stereoisomers of cephalosporolide H is described, made possible by a zinc-chelation strategy for controlling the stereochemistry of oxygenated 5,5-spiroketals. The same strategy likewise enables the first stereocontrolled synthesis of cephalosporolide E, which is typically isolated and prepared admixed with its spiroketal epimer, cephalosporolide F.

## Introduction

The spiroketal moiety is common in natural products of marine, plant, insect, and bacterial origins [1–11]. The rigidity of the spiroketal provides defined orientation of pendant functional groups, and there is a strong correlation between bioactivity and spiroketal stereochemistry in many natural spiroketals. For example, cephalostatin and ritterazine feature thermodynamically disfavored spiroketals that are more cytotoxic than their stereoisomers [12]. Other prominent cytotoxic spiroketals include spongistatin [7] and norhalichondrin [13–14].

**Figure 1:**
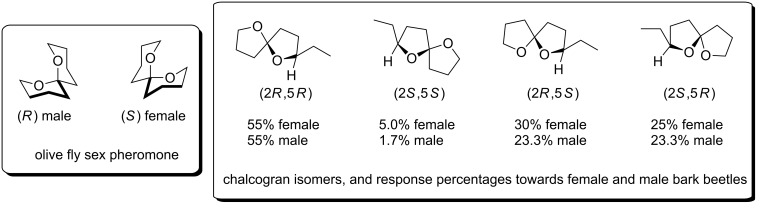
Pheromone spiroketals.

Spiroketal-containing pheromones are especially prominent in insect communication, with the spiroketal stereochemistry often relaying important information [15]. For example, the *R*-enantiomer of the olive-fly sex pheromone is attractive to males, while the *S*-enantiomer attracts females (Figure 1). Chalcogran, a sex pheromone secreted by the male bark beetle, is isolated from natural sources as a mixture of diastereomers. However, Byers et al showed that the spiroketal (2*R*,5*R*)-isomer induces the strongest responses from both females and males [16]. In such cases it is interesting to conjecture that the natural pheromone signal may include a “time stamp”: release of a stereodefined, nonthermodynamic and labile spiroketal enables the detecting insect to gauge the age of the message based on the integrity of the spiroketal center. To duplicate such communication (e.g., for insect population control) requires the ability to prepare specific spiroketal stereoisomers. Anomeric effects typically guide the stereochemical preferences in 6,6 and 5,6-spiroketals [7,17–20], whereas 5,5-spiroketal stereochemistry is more difficult to predict [21] and control [22–25].

**Figure 2:**
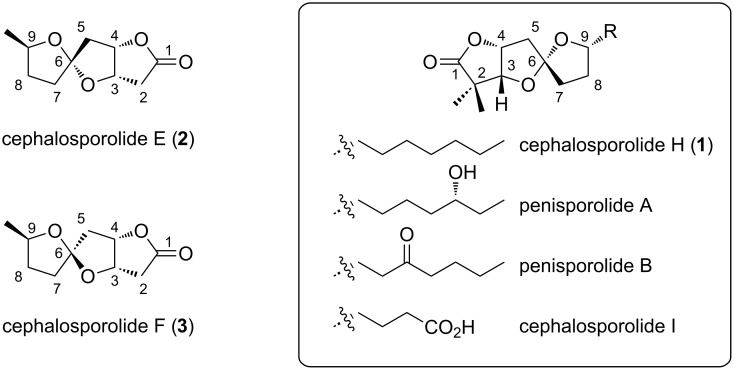
Reported structures of the cephalosporolides and penisporolides.

This report focuses on 5,5-spiroketal lactones of the cephalosporolide and related families (Figure 2) [26–27]. Cephalosporolides E and F are co-isolated as a mixture, and previous syntheses likewise produce these compounds as a mixture in the absence of methods to control the spiro-center [28–30]. However, it is quite possible that the microorganism produces one or the other of these isomers selectively, and that this material scrambles over time to a thermodynamic mixture. It is this mixture that is ultimately extracted, and if this conjecture is true, then the natural extract (mixture) would misrepresent the compound as used in the biological system. Cephalosporolide H, I, and the penisporolides, on the other hand, were isolated as single isomers [31]. The structures of these related spiroketals were tentatively proposed based on NOESY experiments, and by analogy to each other and to the confirmed structures of cephalosporolide E and F.

## Results and Discussion

**Scheme 1:**
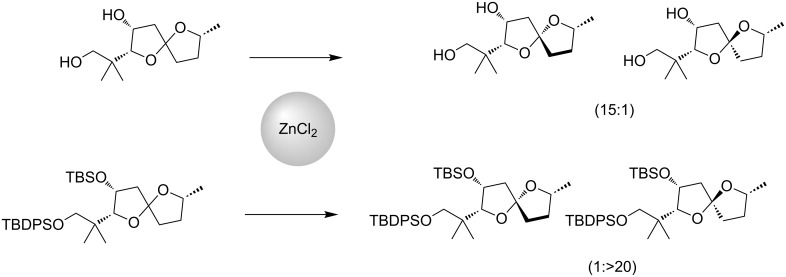
Stereocontrol of oxygenated 5,5-spiroketals.

We recently reported the stereocontrolled synthesis of cephalosporolide H (reported structure) and its spiroketal isomer [32]. Our strategy featured the use of zinc salts to control the spiro-center using either steric biases or chelation, depending on pendant functionality (Scheme 1). Unfortunately, neither of the synthetic isomers provided data in complete agreement with data reported for the natural material.

Here, we report the synthesis of all four possible diastereomers with respect to C9 and the spiroketal center (C6). Data from this study allows us to comment on the proposed structure for cephalosporolide H and, by analogy, cephalosporolide I and the penisporolides. Expanding on the zinc chelation method, we investigated a new approach to the synthesis of cephalosporolide E, which resulted in the first stereocontrolled assembly of this spiroketal core.

**Scheme 2:**
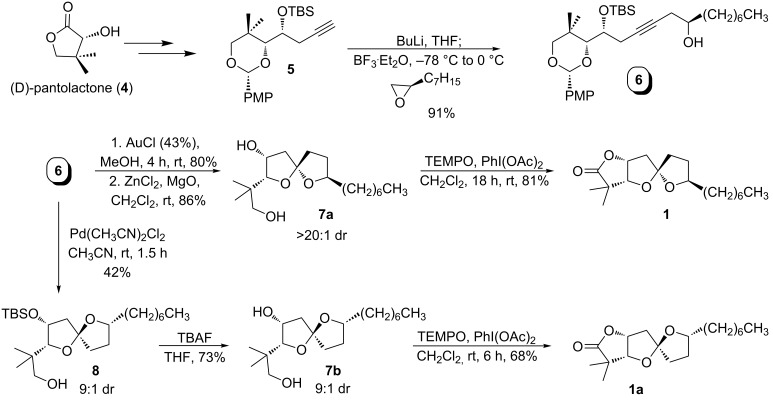
Synthesis of the reported cephalosporolide H and its spiro isomer.

Our synthesis of the reported structure of cephalosporolide H (**1**) and its spiroketal isomer (**1b**) are outlined in Scheme 2. The synthesis starts from D-pantolactone (**4**), which was converted into terminal alkyne **5** [32]. Alkyne **5** was coupled with (*R*)-1,2-epoxynonane to obtain internal alkyne **6**, which was submitted to gold-catalyzed cycloisomerization [33] to afford spiroketals **7a** and **7b** (the silyl ether is concomitantly hydrolyzed) as a 1:1 mixture of isomers. Exposure of this mixture to zinc chloride promoted isomerization to provide **7a** in >20:1 dr. TEMPO oxidation then completed the synthesis of **1**, the reported structure of cephalosporolide H. The opposite spiroketal isomer **1a** was prepared from **6** by palladium-catalyzed cycloisomerization (steric control), desilylation and TEMPO oxidation. In neither case did the characterization data match that of the natural material, and it is worth noting that NOE and NOESY experiments conducted on both isomers were inconclusive in attempts to differentiate them: similar NOESY cross-peaks were observed from both isomers.

**Scheme 3:**
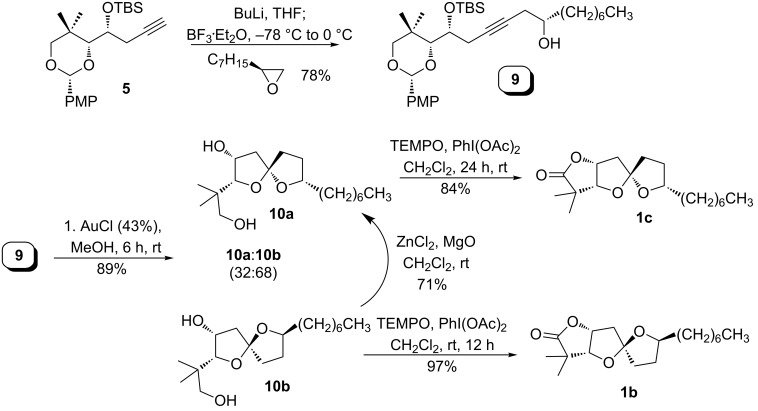
Synthesis of the reported C9-*epi*-cephalosporolide H and its spiro isomer.

We prepared the two remaining core diastereomers by similar routes (Scheme 3), starting by coupling terminal alkyne **5** with (*S*)-1,2 epoxynonane. Gold-catalyzed cycloisomerization (with desilylation) provided spiroketal diols **10a** and **10b** in a 32:68 ratio and in 89% total yield. Major spiroketal **10b** could be converted to **10a** in 15:1 dr by zinc-catalyzed isomerization. Both isomers (**10a** and **10b**) were independently subjected to TEMPO oxidation to afford spiroketal lactones **1b** and **1c**. Spectroscopic data of **1b** and **1c** were similar to **1a** and **1**, respectively.

**Figure 3:**
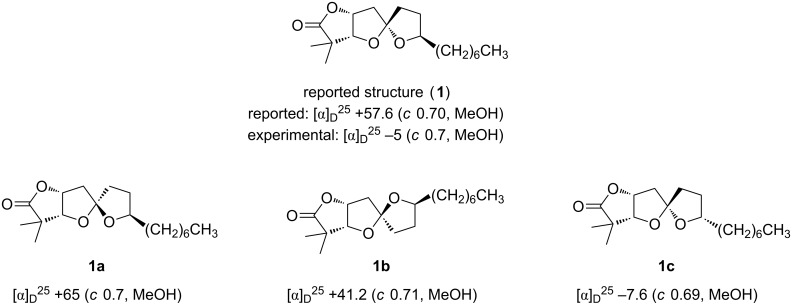
Reported and synthesized cephalosporolide H isomers.

Attempts to secure an authentic sample and/or copies of original NMR spectra for the natural material were unsuccessful, but two candidates emerged as good fits to the reported data (Figure 3). Spectroscopic data for **1a** and **1b** were both nearly consistent with the data reported for cephalosporolide H, whereas data for **1** and **1c** did not match (see Supporting Information File 1, Table S1 for a full comparison). In the absence of an authentic sample, a definitive assignment will not be possible, although we note that the relative stereochemistry of **1b** corresponds to that of cephalosporolide F, the structure of which has been confirmed.

A central feature of this study is our ability to prepare the four diastereomers selectively. Specifically, we found that chelation of zinc salts between the spiroketal oxygen and appropriately positioned hydroxyls overrides normal steric biases to guide the formation of the spiroketal. Cephalosporolide E was targeted for validation of this approach. There are three main differences between cephalosporolides E and H (Figure 2): (1) Cephalosporolide E was isolated (and has been prepared [28]) admixed with cephalosporolide F, whereas cephalosporolide H was isolated as single isomer. (2) Cephalosporolide E (and F) has a C2 methylene; C2 of cephalosporolide H is quaternary. (3) Cephalosporolide E (and F) has a methyl group at C9, as opposed to the *n*-heptyl chain in cephalosporolide H.

**Scheme 4:**
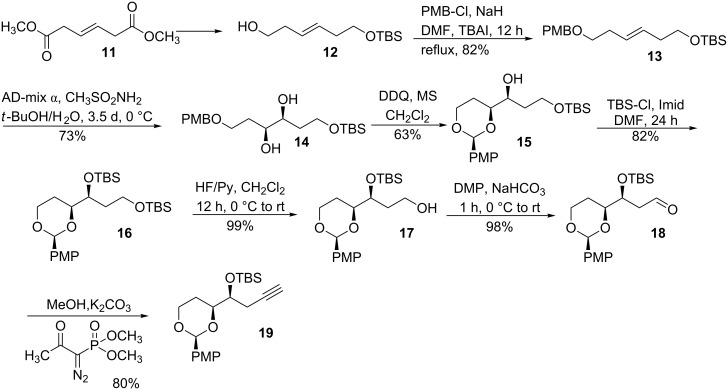
Synthesis of homopropargyl silyl ether.

Synthesis of cephalosporolide E started with the known alcohol **12**, which was prepared from the commercially available diester **11** (Scheme 4) [34]. PMB protection of alcohol **12** followed by Sharpless dihydroxylation afforded diol **14** [35–36]. DDQ oxidation of PMB ether produced 1,3-dioxane **15** [37]. Protecting group manipulation led to the formation of primary alcohol **17** [38], which was converted into homopropargyl silyl ether **19** over two steps, i.e., DMP oxidation and subsequent Ohira–Bestmann ethynylation [39].

**Scheme 5:**
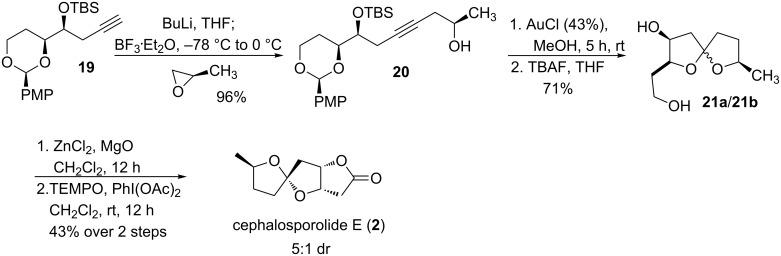
Synthesis of cephalosporolide E.

Coupling of the propargyl silyl ether **19** with the (*R*)-propylene oxide produced the internal alkyne **20** (Scheme 5). Gold(I) chloride in MeOH induced the spiroketalization of alkyne **20** with concomitant cleavage of the PMP acetal and partial cleavage of the TBS ether. After completion of the desilylation with TBAF, a mixture of 5,5-spiroketals **21a** and **21b** was obtained in 71% overall yield from **20**. The mixture of diols **21a** and **21b** converged to **21a** (epimer **21b** no longer observable by ^1^H NMR) upon treatment with zinc chloride. TEMPO oxidation of diol **21a** led to the formation of cephalosporolide E (**2**, admixed with a minor diastereomer tracing back to the Sharpless dihydroxylation reaction). Spectroscopic data for our synthetic material was in full agreement with the reported data for cephalosporolide E [28–30].

## Conclusion

We have completed the stereocontrolled synthesis of the reported structure of cephalosporolide H and three diastereoisomers, leading us to suggest a potential structure for natural cephalosporolide H (i.e., **1b**, or perhaps **1a**). Chelation by using zinc chloride plays a key role in accessing the otherwise contra-thermodynamic spiroketal stereoisomers. This strategy was expanded to enable synthetic production of cephalosporolide E for the first time in a stereocontrolled manner.

## Supporting Information

File 1 General experimental procedures, experimental and characterization data for new compounds, copies of NMR spectra, and comparison of characterization data reported for cephalosporolide H and obtained for the four synthetic diastereomers reported here.
