# Supplementary material for: On the proposed structures and stereocontrolled synthesis of the cephalosporolides
Source: Beilstein J Org Chem. 2012 Aug 14;8:1287–92. doi: 10.3762/bjoc.8.146 (PMC3458751; doi:10.3762/bjoc.8.146)
Supplement: File 1 — General experimental procedures, experimental and characterization data for new compounds, copies of NMR spectra, and comparison of characterization data reported for cephalosporolide H and obtained for the four synthetic diastereomers reported here. [file Beilstein_J_Org_Chem-08-1287-s001.pdf]

Supporting Information

for

On the proposed structures and stereocontrolled synthesis of the cephalosporolides

Sami F. Tlais and Gregory B. Dudley*

Address: Department of Chemistry and Biochemistry, Florida State University, Tallahassee, FL 32306-4390 USA, Fax: (850) 644-8281

Email: Gregory B. Dudley - gdudley@chem.fsu.edu

* Corresponding author

General experimental procedures, experimental and characterization data for new compounds, copies of NMR spectra, and comparison of characterization data reported for cephalosporolide H and obtained for the four synthetic diastereomers reported here.

Contents

General information S2

Experimental S2

^{1}H NMR and ^{13}C NMR spectra S15

References S36

General information

^{1}H NMR and ^{13}C NMR spectra were recorded by using $CDCl_3$ as the deuterated solvent. The chemical shifts (δ) are reported in parts per million (ppm) relative to the residual $CHCl_3$ peak (7.26 ppm for ^{1}H NMR and 77.0 ppm for ^{13}C NMR for all compounds with TMS as internal reference). The coupling constants (*J*) are reported in hertz (Hz). IR spectra were recorded on a FT-IR spectrometer (100). Mass spectra were recorded by using electron ionization (EI) or fast-atom bombardment (FAB). Melting points are uncorrected. Yields refer to isolated material judged to be ≥95% pure by ^{1}H NMR spectroscopy following silica gel chromatography. All chemicals were used as received unless otherwise stated. All solvents, solutions and liquid reagents were added via syringe. Tetrahydrofuran (THF) was purified by distillation over sodium and benzophenone. Methylene chloride (CH_2Cl_2) was distilled from calcium hydride (CaH_2). Methanol (MeOH) and acetonitrile (CH_3CN) were used without any purification. The *n*-BuLi solutions were titrated against a known amount of menthol dissolved in tetrahydrofuran, by using 1,10-phenanthroline as the indicator at 0 °C. All reactions were carried out under an inert nitrogen atmosphere, unless otherwise stated. The purifications were performed by flash chromatography on silica gel F-254 (230–499 mesh particle size).

Experimental

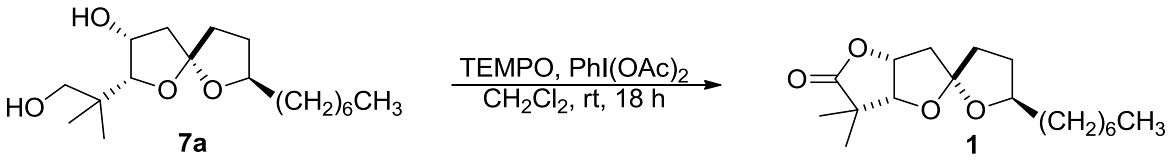

[α] −5 (*c* 0.7, MeOH) (*reported value for cephalosporolide H:*[α] +57.6); ^{1}H NMR (400 MHz, $CDCl_3$) δ 5.07 (t, *J* = 4.9 Hz, 1H), 4.29 (d, *J* = 4.7 Hz, 1H), 4.03–3.92 (m, 1H), 2.44 (d, *J* = 14.1 Hz, 1H), 2.15–1.95 (m, 4H), 1.75–1.59 (m, 2H), 1.29 (s, 3H), 1.25 (br m, 11H), 1.21 (s, 3H), 0.87 (t, *J* = 6.9 Hz, 3H); ^{13}C NMR (100 MHz, $CDCl_3$) δ 180.6, 115.1, 87.1, 81.9, 79.8, 44.5, 41.7, 37.4, 36.3, 31.8, 30.9, 29.6, 29.2, 26.1, 24.9, 22.7, 18.3, 14.1; IR (Neat): 2998, 2927, 2856, 1774, 1457, 1439; HRMS (ESI^+): $[M + Na^+]^+$ calcd. for $C_{18}H_{30}O_4Na$, 333.2042; found, 333.2036.

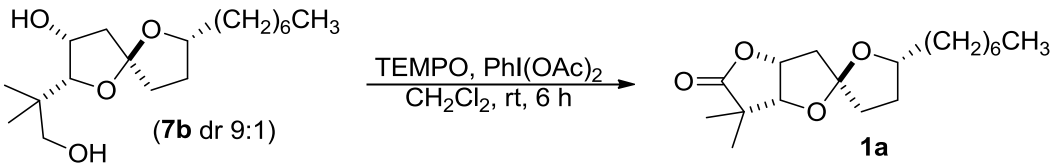

$[\alpha]_D^{25}$ +65 (*c* 0.7, MeOH) (*reported value for cephalosporolide H*: $[\alpha]_D^{25}$ +57.6)**;** ^{1}H NMR (400 MHz, $CDCl_3$) δ 5.03 (ddd, *J* = 6.4, 3.8, 1.6 Hz, 1H), 4.29 (d, *J* = 3.8 Hz, 1H), 4.09–3.98 (m, 1H), 2.52 (dd, *J* = 15.0, 6.4 Hz, 1H), 2.36 (dd, *J* = 15.0, 1.5 Hz, 1H), 2.18–1.97 (m, 3H), 1.55–1.47 (m, 1H), 1.27 (s, 12H), 1.22 (s, 3H), 0.88 (t, *J* = 6.9 Hz, 3H); ^{13}C NMR (100 MHz, $CDCl_3$) δ 181.0, 115.5, 85.2, 80.6, 79.0, 44.5, 42.4, 35.7, 35.5, 31.8, 30.1, 29.6, 29.2, 25.8, 23.1, 22.7, 18.1, 14.1; IR (neat): 2926, 2856, 1778, 1462, 1390; HRMS (ESI^+): $[M + Na^+]^+$ calcd. for $C_{18}H_{30}O_4Na$, 333.2042; found, 333.2033.

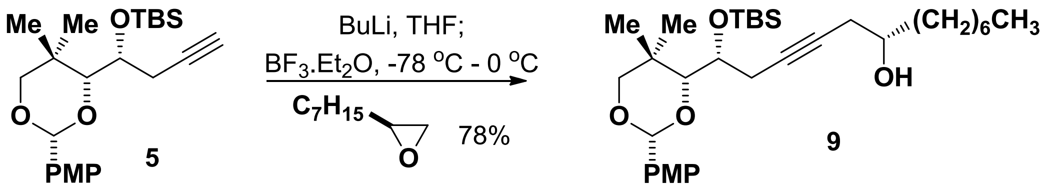

To a clear solution of alkyne **5** (565 mg, 1.4 mmol) and THF (26 mL) at −78 °C under a nitrogen atmosphere, *n*-BuLi (2.5 M in hexanes, 1.68 equiv) was added. The solution immediately turned yellow in color, and after 1 h, freshly distilled neat $BF_3{\cdot}Et_2O$ (0.19 mL, 1.54 mmol) was added. After 6 min, dry and neat (*S*)-1,2 epoxynonane [1] (200 mg, 1.4 mmol) was added. The yellow solution was stirred at −78 °C to 0 °C over 6 h. The reaction was quenched with H_2O (10 mL) and extracted with 40 mL of EtOAc, and the aqueous layer was mixed with saturated NH_4Cl (20 mL) and extracted with 10 mL of EtOAc. The combined organic layers was washed with 20 mL of brine solution, dried under $MgSO_4$ and concentrated under reduced pressure to afford a crude oil, which was purified over silica gel (20% EtOAc in hexane) to produce yellowish oil **9** (596 mg, 78%).

^{1}H NMR (600 MHz, $CDCl_3$) δ 7.44 (d, *J* = 8.6 Hz, 2H), 6.88 (d, *J* = 8.6 Hz, 2H), 5.41 (s, 1H), 4.00–3.94 (m, 1H), 3.81 (s, 3H), 3.73–3.65 (m, 1H), 3.64 (d, *J* = 4.1 Hz, 1H), 3.57 (q, *J* = 11.0 Hz, 2H), 2.66 (dd, *J* = 16.3, 7.2 Hz, 1H), 2.44–2.22 (m, 3H), 1.99–1.85 (m, 1H), 1.52 (dd, *J* = 14.1, 6.7 Hz, 1H), 1.48–1.20 (m, 15H), 0.95–0.79 (m, 16H), 0.05 (s, 3H), –0.02 (s, 3H); ^{13}C NMR (150 MHz, $CDCl_3$) δ 159.9, 131.4, 127.7, 113.5, 102.4, 85.3, 80.2, 80.1, 78.3, 71.3, 70.2, 55.3, 36.3, 32.8, 31.8,

31.6, 29.7, 29.3, 27.8, 25.9, 25.7, 25.3, 22.6, 22.1, 20.2, 18.1, 14.1, −4.1, −4.2; HRMS (ESI$^+$): $[M + Na^+]^+$ calcd for $C_{32}H_{54}O_5SiNa$, 569.3638; found, 569.3616.

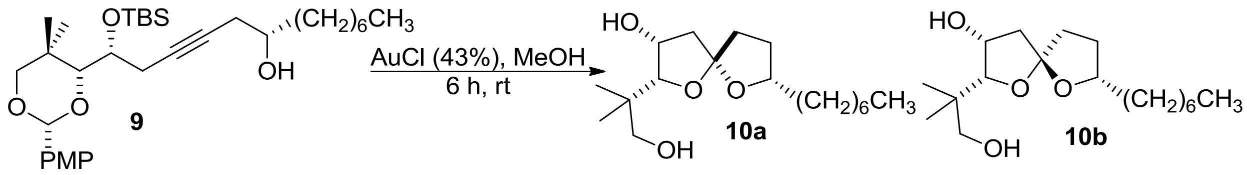

AuCl (9 mg, 0.03 mmol) was added to premixed **9** (50 mg, 0.091 mmol) and MeOH (10 mL) at rt, and a black coloring instantly appeared. After 6.5 h, the reaction mixture was quenched with Et_3N (0.2 mL). After solvent evaporation under reduced pressure, the crude mixture was transferred into silica gel column (15% EtOAc in hexane) to afford pure product **10a** (8 mg) and **10b** (17 mg) (25 mg, 89%).

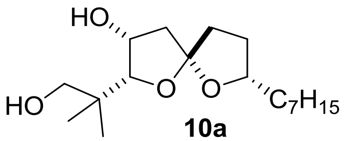

10a

^{1}H NMR (600 MHz, $CDCl_3$) δ 4.31–4.24 (m, 1H), 4.18–4.10 (m, 1H), 3.67 (d, J = 3.3 Hz, 1H), 3.50 (d, J = 10.9 Hz, 1H), 3.47 (d, J = 10.9 Hz, 1H), 2.17 (dd, J = 13.3, 4.3 Hz, 1H), 2.14–1.97 (m, 4H), 1.55–1.22 (m, 13H), 1.08 (s, 3H), 1.03 (s, 3H), 0.88 (t, J = 7.1 Hz, 3H); ^{13}C NMR (150 MHz, $CDCl_3$) δ 114.2, 90.8, 79.0, 73.7, 71.3, 43.9, 38.1, 35.6, 34.3, 31.8, 29.6, 29.6, 29.2, 26.0, 23.0, 22.6, 21.0, 14.1.

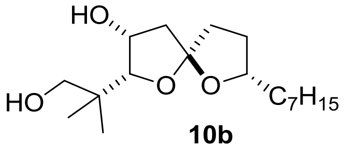

10b

^{1}H NMR (400 MHz, $CDCl_3$) δ 4.35 (dd, J = 4.4, 2.9 Hz, 1H), 4.10–3.97 (m, 1H), 3.69–3.58 (m, 2H), 3.43 (d, J = 10.6 Hz, 1H), 2.99 (br s, 2H), 2.44 (dd, J = 14.4, 5.6 Hz, 1H), 2.24–1.87 (m, 4H), 1.85–1.70 (m, 1H), 1.26 (s, 12H), 1.05 (s, 3H), 1.04 (s, 3H), 0.87 (t, J = 6.8 Hz, 3H); ^{13}C NMR (100 MHz, $CDCl_3$) δ 112.8, 87.4, 79.9, 72.6, 69.4, 45.9, 37.8, 37.7, 37.4, 31.8, 30.8, 29.6, 29.3, 26.0, 24.1, 24.1, 22.6, 21.8, 14.1; mixture of both isomers (**10a** and **10b**), HRMS (ESI$^+$): $[M + Na^+]^+$ calcd for $C_{18}H_{34}O_4Na$, 337.2354; found, 337.2359.

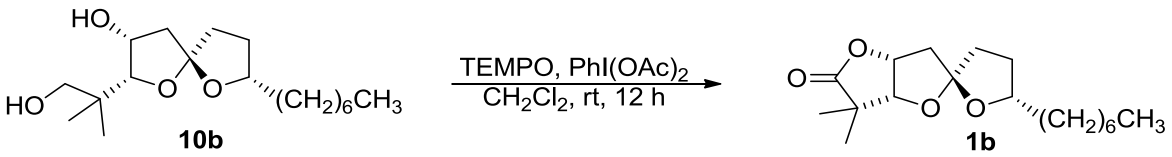

Diol **10b** (16 mg, 0.05 mmol) and $PhI(OAc)_2$ (80 mg, 0.25 mmol) were dissolved in CH_2Cl_2 (5 mL) followed by the addition of TEMPO (5.3 mg, 0.034 mmol). After 12 h, the mixture was concentrated, followed by column chromatography (10% EtOAc in hexane), which afforded lactone **1b** (15 mg, 97%). $[\alpha]_D^{25}$ +41.2 (*c* 0.71, MeOH) (*reported value for cephalosporolide H*: + 57.6); ^{1}H NMR (400 MHz, $CDCl_3$) δ 5.02 (ddd, *J* = 6.3, 3.8, 1.6 Hz, 1H), 4.30 (d, *J* = 3.8 Hz, 1H), 4.07–3.95 (m, 1H), 2.49 (dd, *J* = 15.0, 6.3 Hz, 1H), 2.33 (dd, *J* = 15.0, 1.5 Hz, 1H), 2.11–1.87 (m, 4H), 1.79–1.67 (m, 1H), 1.28 (br m, 12H), 1.26 (s, 3H), 1.22 (s, 3H), 0.88 (t, *J* = 6.9 Hz, 3H); ^{13}C NMR (100 MHz, $CDCl_3$) δ 181.0, 115.2, 85.0, 80.9, 80.4, 44.4, 41.8, 37.2, 37.0, 31.8, 30.6, 29.6, 29.2, 26.0, 23.1, 22.6, 18.1, 14.1; IR (neat): 2927, 2856, 1778, 1460, 1205, 1124; HRMS (ESI$^+$): $[M + Na^+]^+$ calcd for $C_{18}H_{30}O_4Na$, 333.2042; found, 333.2021.

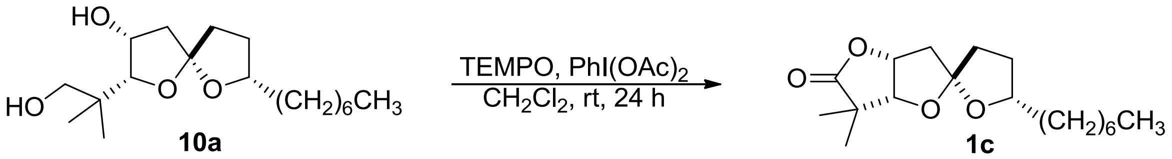

Diol **10a** (6 mg, 0.019 mmol) and $PhI(OAc)_2$ (30 mg, 0.093 mmol) were dissolved in CH_2Cl_2 (2 mL) followed by the addition of TEMPO (2 mg, 0.013 mmol). After 24 h, the mixture was concentrated, followed by column chromatography (10% EtOAc in hexane), which afforded lactone **1c** (5.0 mg, 84%). [α] –7.6 (*c* 0.69, MeOH) (*reported value for cephalosporolide H*: + 57.6); ^{1}H NMR (400 MHz, $CDCl_3$) δ 5.10 (t, *J* = 5.2 Hz, 1H), 4.35 (d, *J* = 5.1 Hz, 1H), 4.16–3.90 (m, 1H), 2.45 (d, *J* = 14.1 Hz, 1H), 2.18–1.96 (m, 4H), 1.54–1.25 (m, 13H), 1.25 (s, 3H), 1.22 (s, 3H), 0.87 (t, *J* = 6.9 Hz, 3H); ^{13}C NMR (100 MHz, $CDCl_3$) δ 180.8, 114.9, 86.7, 79.9, 79.6, 44.3, 41.9, 35.3, 34.5, 31.9, 29.9, 29.6, 29.2, 25.9, 25.4, 22.7, 18.4, 14.1; IR (neat): 2927, 2857, 1775, 1460, 1345, 1304; HRMS (CI$^+$): $[M + H^+]^+$ calcd for $C_{18}H_{31}O_4$, 311.2222; found, 311.2217.

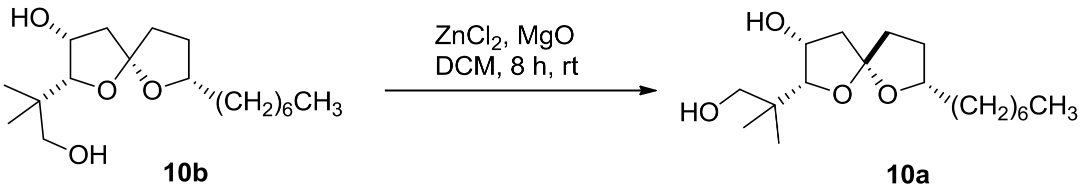

10b **10a**

Diol **10b** (7 mg, 0.022 mmol), $ZnCl_2$ (16 mg, 0.12 mmol), and acid scavenger MgO (100 mg, 0.5 mmol) were mixed with dry CH_2Cl_2 (2 mL). After 8 h, the reaction mixture was filtered and then quenched with saturated solution of EDTA (1 mL). The reaction mixture was extracted with CH_2Cl_2 (10 mL), washed with $NaHCO_3$ (5 mL), washed with brine (3 mL), and dried over $MgSO_4$. The reaction mixture was concentrated under reduced pressure and purified over silica gel (deactivated with Et_3N) to afford **10a** (5 mg, 71% yield, 15:1 dr).

Table S1: Comparison between the synthesized isomers and reported ^{1}H NMR and ^{13}C NMR for cephalosporolide H.

reported data for natural cephalosporolide H		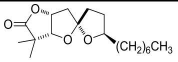 reported structure		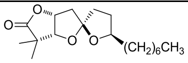 likely candidate		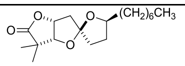 possible candidate		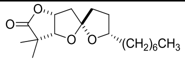			
$[\alpha]_D^{25}$ +57.6		**1** $[\alpha]_D^{25}$ –5		**1a** $[\alpha]_D^{25}$=+ 65		**1b** $[\alpha]_D^{25}$ +41.2		**1c** $[\alpha]_D^{25}$ –7.6		Δδ for **1a** and **1b**	
^{1}H NMR	^{13}C NMR	^{1}H NMR	^{13}C NMR	^{1}H NMR	^{13}C NMR	^{1}H NMR	^{13}C NMR	^{1}H NMR	^{13}C NMR	^{1}H NMR	^{13}C NMR
5.01 (m)	180.8	5.07 (t)	180.6	5.03 (ddd)	181	5.02 (ddd)	181.0	5.10 (t)	180.8	0.01	0.0
4.26 (d)	115.5	4.29 (d)	115.1	4.29 (d, *J* = 3.8	115.5	4.3 (d)	115.2	4.35 (d)	114.9	–0.01	0.3
4.05 (m)	85.2	4.03–3.92 (m)	87.1	4.09–3.98 (m)	82.2	4.07–3.95 (m)	85.0	4.16–3.90 (m)	86.72	–	–2.8
2.52 (d)	80.5	2.44 (d)	82.0	2.52 (dd)	80.6	2.49 (dd)	80.8	2.45 (d)	79.9	0.03	–0.2

2.35 (d)	78.8	2.15–1.95 (m)	79.9	2.36 (dd)	79.0	2.33 (dd)	80.4	2.18–1.96 (m)	79.6	0.03	–1.4
2.2–2.1 (m)	44.4	1.75–1.59 (m)	44.5	2.18 1.97 (m	44.5	2.11–1.87 (m)	44.4	1.76–1.60 (m)	44.3	–	0.1
1.65–1.23 (m)	42.4	1.25 (br m)	41.7	1.55–1.47 (m)	42.3	1.79–1.28 (m)	41.8	1.54–1.25 (br m)	41.9	–	0.5
1.24 (s)	35.5	1.29 (s)	37.4	1.27 (s)	35.7	1.26 (s)	37.2	1.25 (s)	35.3	0.01	–1.5
1.19 (s)	33.8	1.21 (s)	36.3	1.22 (s)	35.5	1.22 (s)	37.0	1.22 (s)	34.5	0.00	–1.5
0.84 (t)	31.6	0.87 (t)	31.8	0.88 (t)	31.8	0.88 (t)	31.8	0.87 (t)	31.9	0.00	0.0
	30.1		30.9		30.1		30.62		29.9		–0.52
	29.2		29.6		29.6		29.6		29.6		0.0
	29.0		29.2		29.2		29.2		29.2		0.0
	25.1		26.1		25.8		26.0		25.9		0.2
	23.1		24.9		23.1		23.1		25.4		0.0
	22.5		22.7		22.7		22.6		22.7		0.1
	18.1		18.3		18.1		18.1		18.4		0.0
	14.0		14.1		14.1		14.1		14.1		0.0

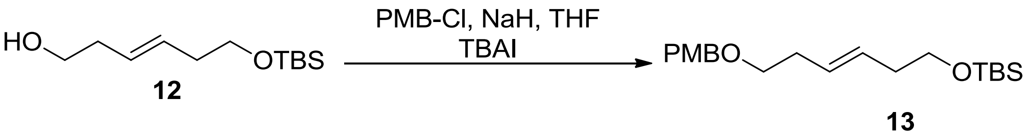

Alcohol **12** (4.27 g, 18.33 mmol) was mixed with NaH (60% in oil, 1.3 g) and 90 mL of THF. After 30 min of stirring at rt, PMB-Cl (5 mL, 37 mmol) and a catalytic amount of TBAI (100 mg) were added. The mixture was heated under reflux for 12 h. The reaction was quenched with 50 mL of H_2O at 0 °C, and extracted with Et_2O (100 mL). After solvent evaporation, the reaction crude was purified using bulb-to-bulb distillation by distilling off the impurities at 120 °C and 0.4 mmHg. After silica-gel filtration, PMB ether **13** resulted as a yellowish oil (3.83 g, 82%). ^{1}H NMR (400 MHz, $CDCl_3$) δ 7.30–7.20 (m, 2H), 6.92–6.78 (m, 2H), 5.48 (apparent t, *J* = 3.7 Hz, 2H), 4.44 (s, 2H), 3.80 (s, 3H), 3.61 (t, *J* = 6.9 Hz, 2H), 3.45 (t, *J* = 7.0 Hz, 2H), 2.31 (dd, *J* = 11.1, 6.8 Hz, 2H), 2.22 (dd, *J* = 11.3, 6.6 Hz, 2H), 0.89 (s, 9H), 0.04 (s, 3H); ^{13}C NMR (100 MHz, $CDCl_3$) δ 159.1, 130.5, 129.2, 128.6, 128.4, 113.7, 113.7, 72.4, 69.7, 63.1, 55.1, 36.3, 33.1, 25.9, 18.3, –5.3. IR (Neat): 2953, 2927, 2855, 1614, 1587, 1513, 1039; HRMS (CI$^+$): [M + H$^+$]$^+$ calcd for $C_{20}H_{35}O_3Si$, 351.2356; found, 351.2352.

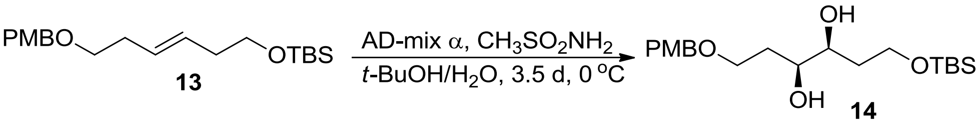

A mixture of AD-mix α (3 g), $CH_3SO_2NH_2$ (285 mg, 3 mmol), *t*-BuOH (10 mL), and H_2O (10 mL) was stirred for 15 min at 0 °C, and then alkene **13** (700 mg, 2.0 mmol) was added. After 1 d of stirring at 0 °C, another 1 g of AD-mix α was added. After 3.5 d as an overall time, $Na_2S_2O_3$ (4 g) and EtOAc (50 mL) were added to the reaction mixture, which was stirred for 30 min at rt. The organic layer was extracted and washed with NH_4Cl and brine solution. Solvant evaporation under reduced pressure afforded a crude oil. The oil was purified with silica gel by using 20% EtOAc in hexane to produce a yellow oil, **14** (562 mg, 73%). ^{1}H NMR (400 MHz, $CDCl_3$) δ 7.29–7.23 (m, 2H), 6.90–6.84 (m, 2H), 5.60–5.29 (m, 2H), 4.44 (s, 2H), 3.80 (s, 3H), 3.61 (t, *J* = 6.9 Hz, 2H), 3.45 (t, *J* = 7.0 Hz, 2H), 2.36–2.26 (m, 2H), 2.25–2.14 (m, 2H), 0.89 (s, 9H), 0.04 (s, 6H); ^{13}C NMR (100 MHz, $CDCl_3$) δ 159.1, 130.5, 129.3, 129.2, 128.6, 128.4, 113.7, 113.7, 72.4, 69.7, 63.1, 55.1, 36.3, 33.1, 25.9, 18.3, –5.3; IR (neat): 3428, 2953, 2856, 1612, 1513, 1082; HRMS (CI$^+$): [M + H$^+$]$^+$ calcd for $C_{20}H_{37}O_5Si$, 385.2410; found, 385.2409.

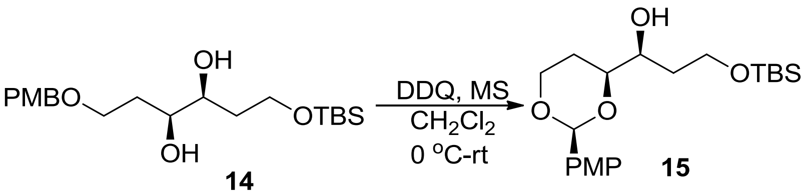

A solution of diol **14** (400 mg, 1.04 mmol) in CH_2Cl_2 (26 mL) and MS (1g) was stirred for 1 h. DDQ (450 mg, 1.47 mmol) was added to the reaction mixture at 0 °C. After 2 h of stirring from 0 °C to rt, the reaction mixture was quenched with 1 M $Na_2S_2O_3$ solution and extracted with diethyl ether (100 mL). The organic layer was washed with saturated $NaHCO_3$ solution and brine, dried with anhydrous sodium sulfate and concentrated in vacuo to provide crude oil, which was purified over silica gel. PMP acetal **15** (254 mg) was isolated in 63% yield. ^{1}H NMR (400 MHz, $CDCl_3$) δ 7.49–7.31 (m, 2H), 7.01–6.74 (m, 2H), 5.49 (s, 1H), 4.29 (ddd, *J* = 11.3, 5.0, 1.2 Hz, 1H), 3.99–3.90 (m, 1H), 3.90–3.81 (m, 2H), 3.80 (s, 3H), 2.97 (d, *J* = 2.8 Hz, 1H), 2.00–1.85 (m, 1H), 1.82–1.50 (m, 3H), 0.90 (s, 9H), 0.07 (s, 6H); ^{13}C NMR (100 MHz, $CDCl_3$) δ 159.9, 131.0, 127.4, 113.6, 113.5, 101.2, 79.9, 72.1, 66.7, 60.7, 55.3, 34.3, 26.7, 25.9, 18.2, –5.4, –5.5; IR (neat): 3489, 2954, 2982, 2856, 1615, 1518, 1091; HRMS (CI$^+$): $[M + H^+]^+$ calcd for $C_{20}H_{35}O_5Si$, 383.2254; found, 383.2246.

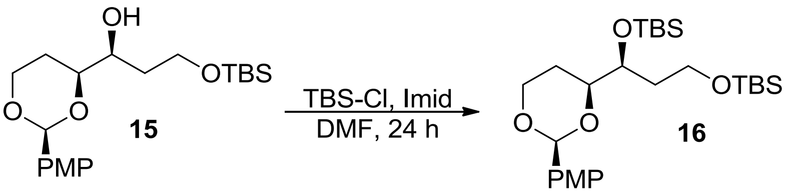

To a magnetically stirred solution of alcohol **15** (230 mg, 0.6 mmol) and imidazole (204 mg, 3.0 mmol) in dry DMF (3 mL), TBSCl (286 mg, 1.88 mmol) was added at rt. After 24 h, the reaction was quenched with H_2O (5 mL) and extracted with Et_2O (30 mL). The organic layer was washed with saturated solution of NH_4Cl, dried over $MgSO_4$, and concentrated under reduced pressure. The crude oil was purified by using silica-gel flash chromatography (1% EtOAc in hexane) to afford silyl ether **16** (245 mg, 82%). ^{1}H NMR (400 MHz, $CDCl_3$) δ 7.43–7.37 (m, 2H), 6.89–6.84 (m, 2H), 5.42 (s, 1H), 4.31–4.22 (m, 1H), 3.97–3.86 (m, 2H), 3.80 (s, 3H), 3.85–3.64 (m, 2H), 1.94–1.75 (m, 2H), 1.69–1.58 (m, 1H), 1.57–1.49 (m, 1H), 0.90 (s, 9H), 0.85 (s, 9H), 0.05 (s, 3H), 0.05 (s, 3H), 0.04 (s, 3H), –0.02 (s, 3H); ^{13}C NMR (100 MHz, $CDCl_3$) δ 159.8, 131.3, 127.6, 113.4, 101.4, 80.3, 71.1, 67.0, 59.8, 55.2, 35.2, 25.9, 25.9, 18.3, 18.2, –4.3, –4.9, –5.2, –5.3; IR (neat): 2954, 2928, 2856, 1616, 1519, 1302; HRMS (CI$^+$): $[M + H^+]^+$ calcd for $C_{26}H_{49}O_5Si_2$, 497.3119; found, 497.3108.

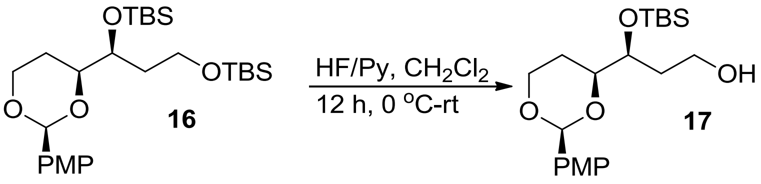

Compound **16** (1.35 g, 2.5 mmol) was dissolved in dry THF (13 mL). The mixture was then treated with a solution of the HF-pyridine complex (0.4 mL, ca. 12.5 mmol of HF). The resulting solution was stirred at rt for 3 h. The reaction mixture was diluted with EtOAc (10 mL). The mixture was slowly quenched at 0 °C with saturated $NaHCO_3$. After extracted of the EtOAc layer, the aqueous layer was extracted with another 10 mL of EtOAc. The combined organic layers was subsequently washed with saturated NH_4Cl and $NaHCO_3$, and dried over $MgSO_4$. After evaporation under reduced pressure, the crude oil was purified with silica gel (elution 20% EtOAc in hexane) to yield alcohol **17** (150 mg, ≥99%). ^{1}H NMR (400 MHz, $CDCl_3$) δ 7.39 (apparent d, *J* = 8.7 Hz, 2H), 6.95–6.79 (m, 2H), 5.44 (s, 1H), 4.29 (dd, *J* = 11.4, 4.0 Hz, 1H), 4.03–3.85 (m, 3H), 3.80 (s, 3H), 3.84–3.71 (m, 2H), 2.26 (t, *J* = 5.4 Hz, 1H), 2.02–1.82 (m, 2H), 1.79–1.67 (m, 1H), 1.52 (dd, *J* = 13.2, 1.4 Hz, 1H), 0.87 (s, 9H), 0.07 (s, 3H), 0.01 (s, 3H); ^{13}C NMR (100 MHz, $CDCl_3$) δ 159.9, 130.9, 127.5, 113.5, 101.7, 80.0, 72.5, 66.9, 59.9, 55.2, 34.5, 25.8, 25.6, 18.0, –4.4, –5.0; IR (neat): 3430, 2954, 2928, 2855, 1615, 1302; HRMS (CI$^+$): [M + H$^+$]$^+$ calcd for $C_{20}H_{35}O_5Si$, 383.2254; found, 383.2265.

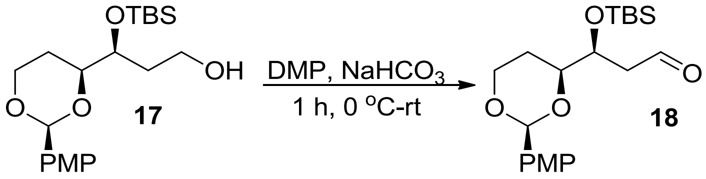

DMP (0.21 mmol, 90 mg) was added to solution of alcohol **17** (40 mg, 0.1 mmol), excess of $NaHCO_3$ (200 mg), and 2 mL of CH_2Cl_2. The reaction mixture was allowed to warm up over 1 h. The mixture was filter over silica gel to afford aldehyde **18** (39 mg, >98%). ^{1}H NMR (400 MHz, $CDCl_3$) δ 9.80 (dd, *J* = 2.9, 1.6 Hz, 1H), 7.42–7.32 (m, 2H), 6.96–6.80 (m, 2H), 5.45 (s, 1H), 4.38 (dt, *J* = 4.8, 7.4 Hz, 1H), 4.29 (dd, *J* = 11.4, 3.9 Hz, 1H), 3.98–3.85 (m, 2H), 3.79 (s, 3H), 2.75 (ddd, *J* = 16.0, 4.5, 1.6 Hz, 1H), 2.56 (ddd, *J* = 16.0, 7.5, 3.0 Hz, 1H), 1.86 (ddd, *J* = 24.8, 12.3, 5.0 Hz, 1H), 1.52 (dd, *J* = 13.2, 1.4 Hz, 1H), 0.94–0.79 (m, 9H), 0.06 (s, 6H); ^{13}C NMR (100 MHz, $CDCl_3$) δ

201.3, 159.9, 130.8, 127.4, 113.5, 101.4, 79.1, 69.1, 66.8, 55.2, 46.6, 25.7, 25.2, 18.0, –4.5, –5.0; IR (neat): 2955, 2856, 1723, 1615, 1518, 1302, 1172; HRMS (CI^+): $[M + H^+]^+$ calcd for $C_{20}H_{33}O_5Si$, 381.2097; found, 381.2091.

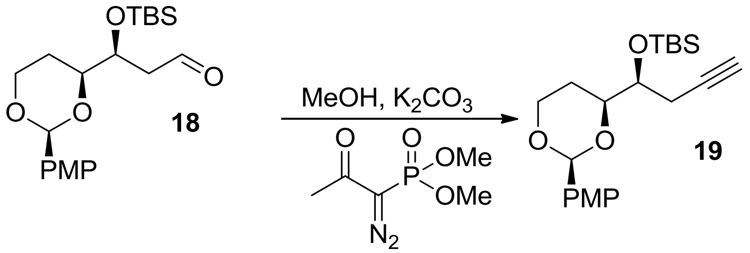

Dimethyl-1-diazo-2-oxopropylphosphonate (Ohira Bestmann) (0.26 mmol, 50 mg) was added to a premixed solution of the aldehyde **18** (0.1 mmol, 40 mg) and K_2CO_3 (0.43 mmol, 60 mg) in 4 mL of MeOH. After 12 h of stirring at rt, the reaction mixture was concentrated and transferred into the column of silica gel. Elution of 1% EtOAc in hexane afforded alkyne **19** (30 mg, 80%). ^{1}H NMR (400 MHz, $CDCl_3$) δ 7.40 (d, *J* = 8.7 Hz, 2H), 6.88 (d, *J* = 8.8 Hz, 2H), 5.46 (s, 1H), 4.28 (dd, *J* = 11.4, 4.0 Hz, 1H), 4.02–3.86 (m, 3H), 3.80 (s, 3H), 2.60 (ddd, *J* = 16.7, 4.4, 2.7 Hz, 1H), 2.34 (ddd, *J* = 16.8, 6.5, 2.7 Hz, 1H), 1.97 (t, *J* = 2.6 Hz, 1H), 1.88 (ddd, *J* = 23.5, 12.5, 5.0 Hz, 1H), 1.56–1.45 (m, 1H), 0.89 (s, 9H), 0.11 (s, 3H), 0.05 (s, 3H); ^{13}C NMR (100 MHz, $CDCl_3$) δ 159.9, 131.1, 127.5, 113.5, 101.4, 81.8, 79.0, 72.5, 69.8, 66.9, 55.2, 25.8, 25.7, 22.7, 18.1, –4.6, –4.6; IR (neat): 3310, 2955, 2928, 2855, 1615, 1518, 1463, 1171; HRMS (CI^+): $[M + H^+]^+$ calcd for $C_{21}H_{33}O_4Si$, 377.2148; found, 377.2152.

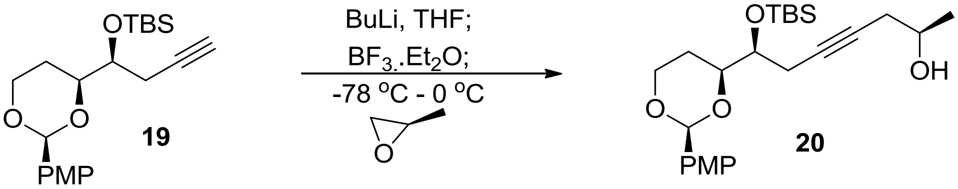

To a clear solution of alkyne **19** (69 mg, 0.18 mmol) and THF (2 mL) at –78 °C under a nitrogen atmosphere, *n*-BuLi (1.8 M in hexanes, 2.7 equiv) was added. The solution immediately turned yellow in color, and after 1 h, freshly distilled neat $BF_3 \cdot Et_2O$ (62 µL, 0.5 mmol) was added. After 6 min, dry and neat excess of (*R*)-propylene oxide (0.1 mL) was added. The yellow solution was stirred at –78 °C to 0 °C over 2.5 h. The reaction was quenched with $NaHCO_3$ (3 mL), and extracted with 10 mL of EtOAc, and the aqueous layer was mixed with saturated NH_4Cl (10 mL) and extracted

with 10 mL of EtOAc. The combined organic layers was washed with 10 mL of brine solution, dried over $MgSO_4$, and concentrated under reduced pressure to afford a crude oil, which was purified over silica gel (15% EtOAc in hexane) to produce a colorless oil **20** (75 mg, 96 %). ^{1}H NMR (400 MHz, $CDCl_3$) δ 7.47–7.31 (m, 2H), 6.96–6.78 (m, 2H), 5.45 (s, 1H), 4.34–4.23 (m, 1H), 3.98–3.83 (m, 4H), 3.80 (s, 3H), 2.63–2.52 (m, 1H), 2.42–2.22 (m, 2H), 1.94–1.80 (m, 1H), 1.55–1.47 (m, 1H), 1.23 (d, J = 6.1 Hz, 3H), 0.89 (s, 9H), 0.09 (s, 3H), 0.04 (s, 3H); ^{13}C NMR (100 MHz, $CDCl_3$) δ 159.9, 131.1, 127.5, 113.5, 101.4, 80.3, 79.3, 77.8, 72.8, 66.9, 66.5, 55.2, 29.5, 25.8, 23.0, 22.2, 18.1, –4.6, –4.6; IR (neat): 3436, 2928, 2855, 1615, 1518, 1372, 937; HRMS (CI^+): $[M + H^+]^+$ calcd for $C_{24}H_{39}O_5Si$, 435.2567; found, 435.2573.

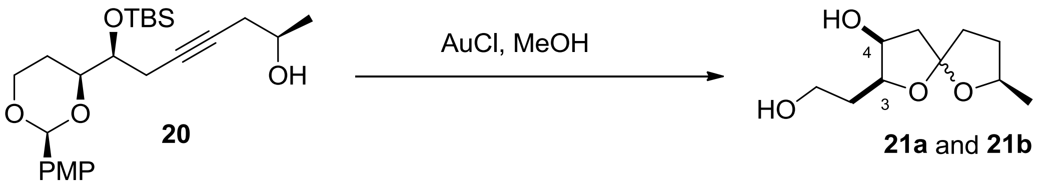

AuCl (6 mg, 0.025mmol) was added to premixed **20** (26 mg, 0.059 mmol) and MeOH (6 mL) at rt, and a black coloring instantly appeared. After 5 h, the reaction mixture was quenched with Et_3N (0.1 mL). After solvent evaporation under reduced pressure, the crude mixture was transferred into a silica gel column (10–70% EtOAc in hexane) to afford **21a** and **21b** in a ratio of 45:55 (5.5 mg) and **22a** and **22b** in a ratio of 16:84. Both isomers admixed with diastereomers from the Sharpless dihydroxylation at C3 and C4.

Mixture of TBS ether isomers **22a** and **22b** were diluted in 0.5 mL of THF and treated with TBAF (1 M, 0.05 mL). After 2 h of stirring at rt, the reaction mixture was concentrated and transferred into a silica-gel column to afford **21a** and **21b** (3 mg) in the same ratio (8.5 mg, 71% overall yield).

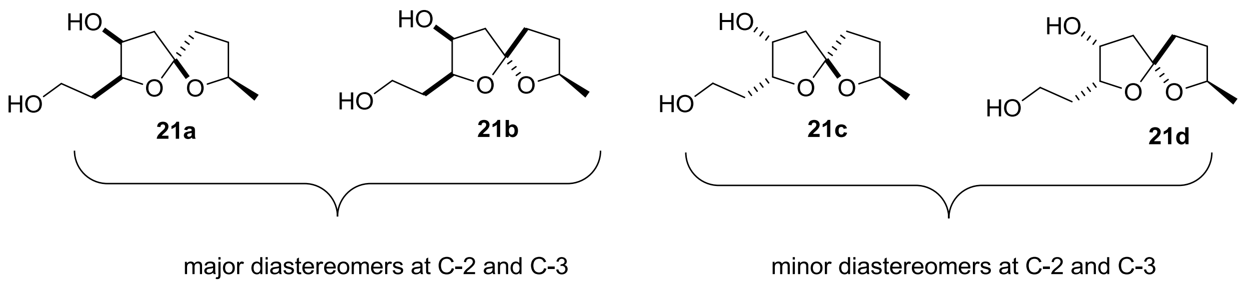

^{1}H NMR (400 MHz, $CDCl_3$) δ 4.41–4.33 (m, 1H), 4.27 (dd, J = 12.8, 6.8 Hz, 1H) [**21b**], 4.23–4.04 (m, 2H), 3.89 (dt, J = 4.9, 10.1 Hz, 1H), 3.82–3.72 (m, 1H), 2.46 (dd, J = 14.3, 6.3 Hz, 1H), 2.23–

1.87 (m, 6H), 1.80–1.67 (m, 1H), 1.31 (d, *J* = 6.0 Hz, 3H) [**21a**], 1.28 (d, *J* = 6.2 Hz, 3H) [**21b**], 1.22 (d, *J* = 6.2 Hz, 3H) [**21c**], 1.20 (d, *J* = 6.2 Hz, 3H) [**21d**]; ^{13}C NMR (100 MHz, $CDCl_3$) δ 114.5, 113.7, 113.6, 83.8, 81.2, 81.0, 77.2, 76.1, 74.8, 74.3, 73.3, 72.5, 60.7, 60.5, 60.3, 45.4, 44.9, 43.8, 37.8, 34.4, 33.1, 32.6, 30.8, 23.0, 21.1, 21.0; IR (neat): 3383, 2926, 1556, 1440, 1335. HRMS (CI^+): $[M + H^+]^+$ calcd for $C_{10}H_{19}O_4$, 202.1283; found, 202.1285.

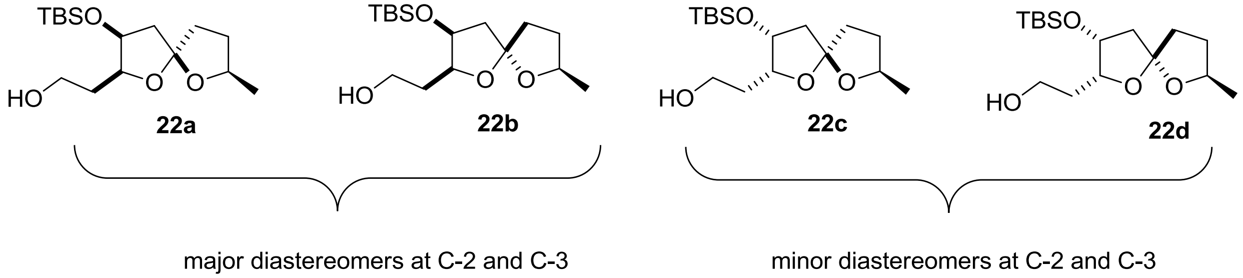

^{1}H NMR (400 MHz, $CDCl_3$) δ 4.35 (dd, *J* = 8.6, 5.0 Hz, 1H), 4.32–4.04 (m, 2H), 3.85–3.72 (m, 1H), 2.63 (s, 1H), 2.33 (dd, *J* = 13.6, 5.8 Hz, 1H), 2.25–1.87 (m, 6H), 1.75–1.61 (m, 1H), 1.49–1.39 (m, 1H) [minor], 1.32 (d, *J* = 6.2 Hz, 3H) [minor], 1.28 (d, *J* = 6.1 Hz, 3H), 1.20 (d, *J* = 6.2 Hz, 3H) [minor], 1.18 (d, *J* = 6.1 Hz, 3H) [minor], 0.89 (s, 9H), 0.05 (d, *J* = 2.6 Hz, 6H); ^{13}C NMR (100 MHz, $CDCl_3$) δ 116.3, 114.0, 113.7, 81.4, 77.2, 76.1, 74.8, 73.1, 72.2, 61.6, 61.4, 45.1, 44.3, 38.3, 37.6, 32.5, 32.0, 25.7, 22.9, 18.0, –4.7, –5.0; IR (neat): 3500, 2929, 836; HRMS (CI^+): $[M + H^+]^+$ calcd for $C_{16}H_{33}O_4Si$, 317.2148; found, 317.2140.

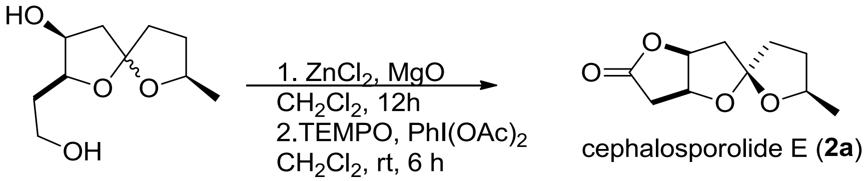

Diol **21a** and **21b** (4 mg, 0.019 mmol), $ZnCl_2$ (10 mg, 0.075 mmol) and acid scavenger MgO (100 mg, 0.5 mmol) were mixed with dry CH_2Cl_2 (1 mL). After 12 h, the reaction mixture was filtered and quenched with saturated solution of $NaHCO_3$. The reaction mixture was extracted with EtOAc (10 mL) and dried over $MgSO_4$. The reaction mixture was concentrated under reduced pressure to afford crude **21a** (3 mg, dr >20:1). The crude **21a** was subjected to oxidative conditions, i.e., $PhI(OAc)_2$ (30 mg, 0.093 mmol) and CH_2Cl_2 (1.5 mL) followed by the addition of TEMPO (2 mg, 0.013 mmol). After 12 h, the mixture was concentrated, followed by column chromatograph

(20% EtOAc in hexane), which afforded lactone **2** (5:1 dr) (1.7 mg, 43% over two steps). The spectroscopic data was in agreement with the reported data. ^{1}H NMR (600 MHz, $CDCl_3$) δ 5.15 (t, *J* = 5.9 Hz, 1H), 4.91–4.86 (m, 1H), 4.23–4.15 (m, 1H), 2.77–2.69 (m, 1H), 2.66 (dd, *J* = 18.6, 1.6 Hz, 1H), 2.45 (d, *J* = 14.3 Hz, 1H), 2.16–2.00 (m, 5H), 1.49–1.40 (m, 3H), 1.20 (d, *J* = 6.2 Hz, 4H); ^{13}C NMR (150 MHz, $CDCl_3$) δ 115.1, 83.3, 77.4, 75.1, 41.8, 37.6, 34.3, 31.4, 29.7, 20.9; IR (neat): 2928, 1777, 1459, 1347; HRMS (CI^+): $[M + H^+]^+$ calcd for $C_{10}H_{15}O_4$, 199.0970; found, 199.0966.

^{1}H NMR and ^{13}C NMR spectra

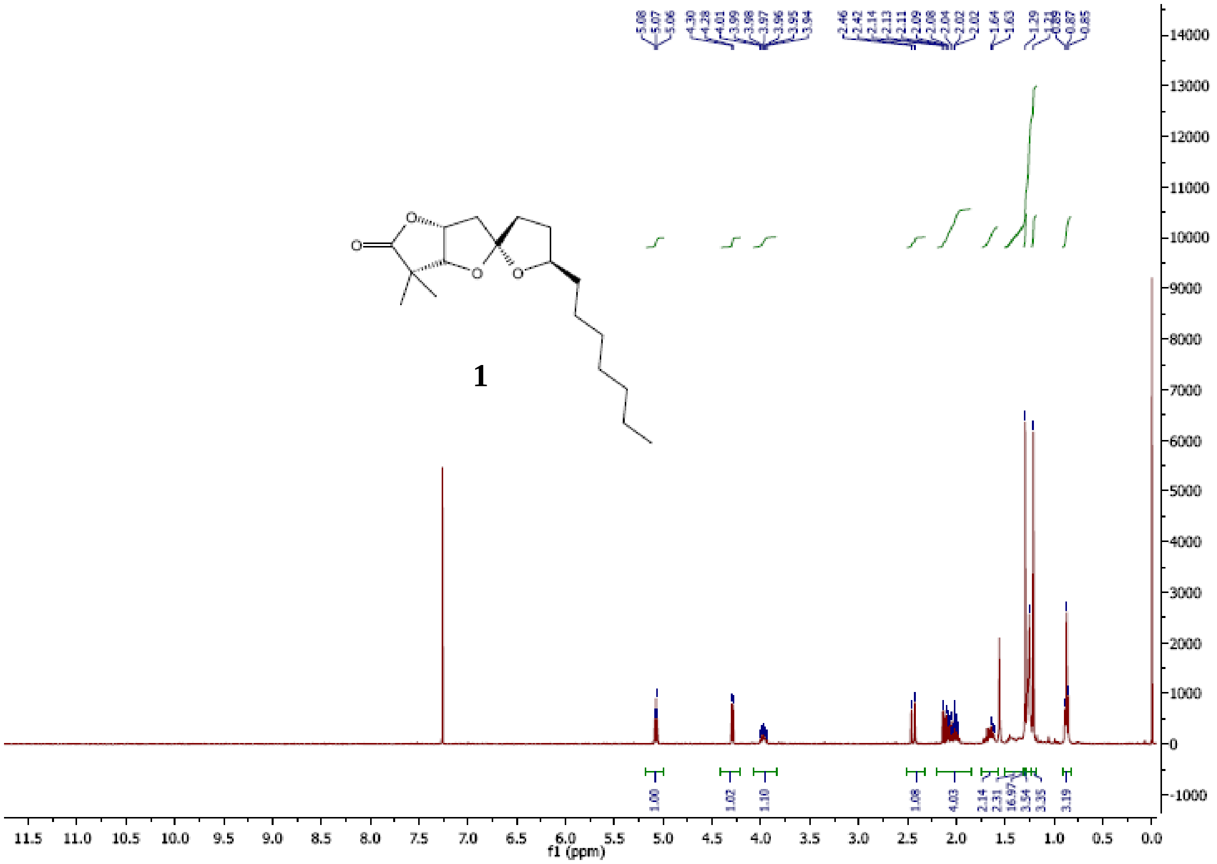

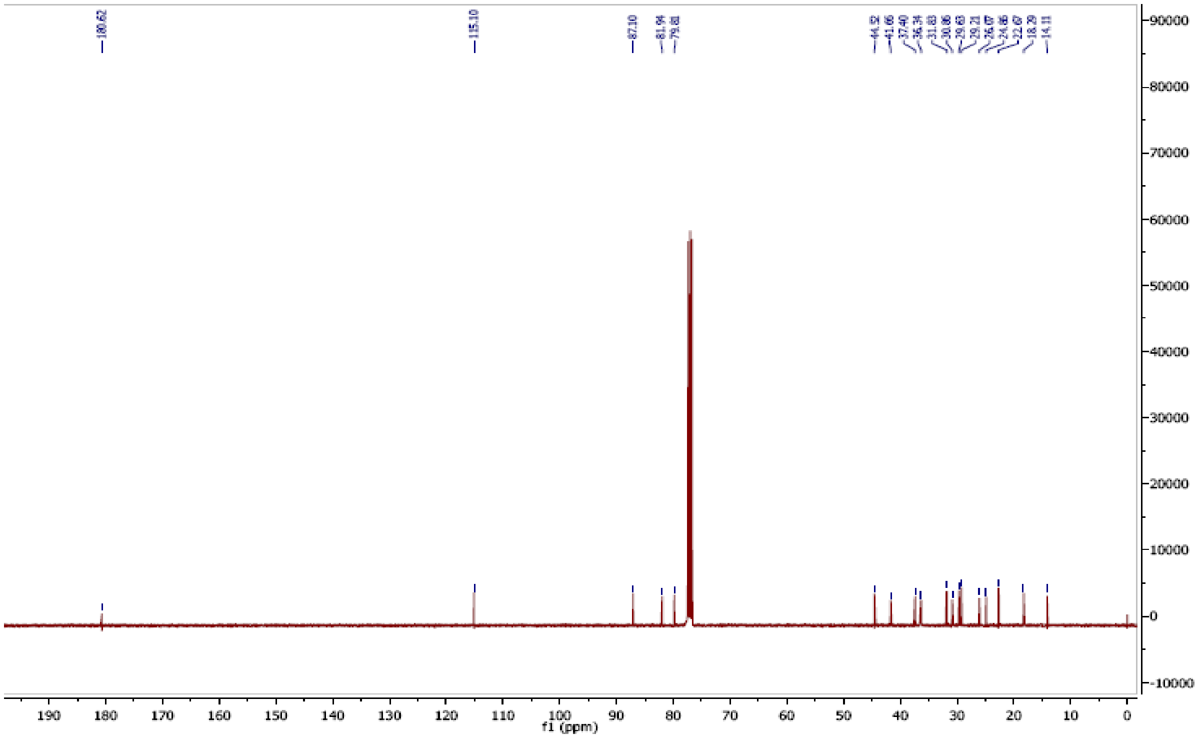

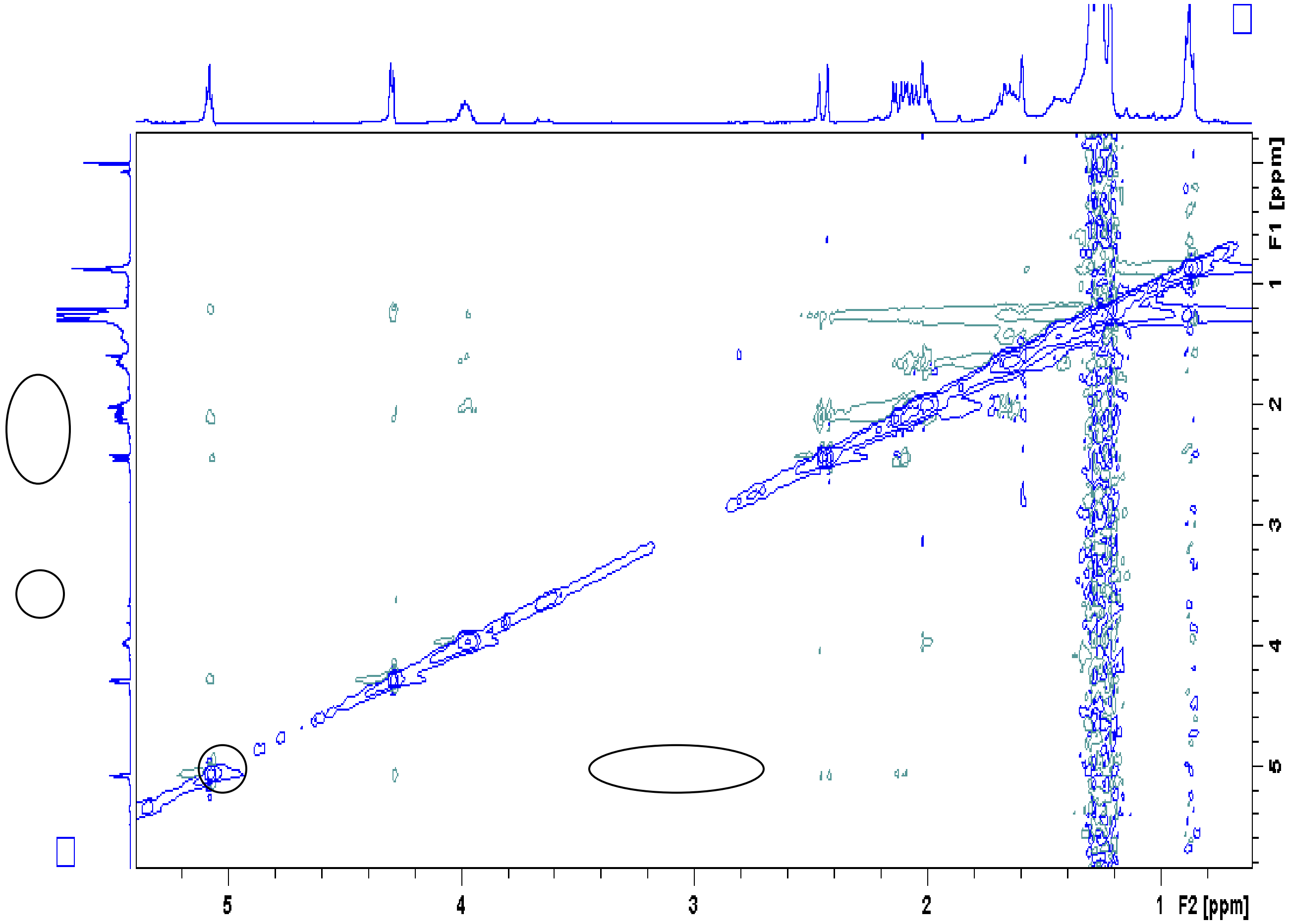
F1 [ppm]
F2 [ppm]

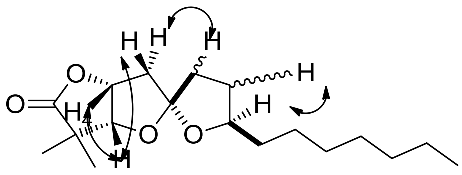

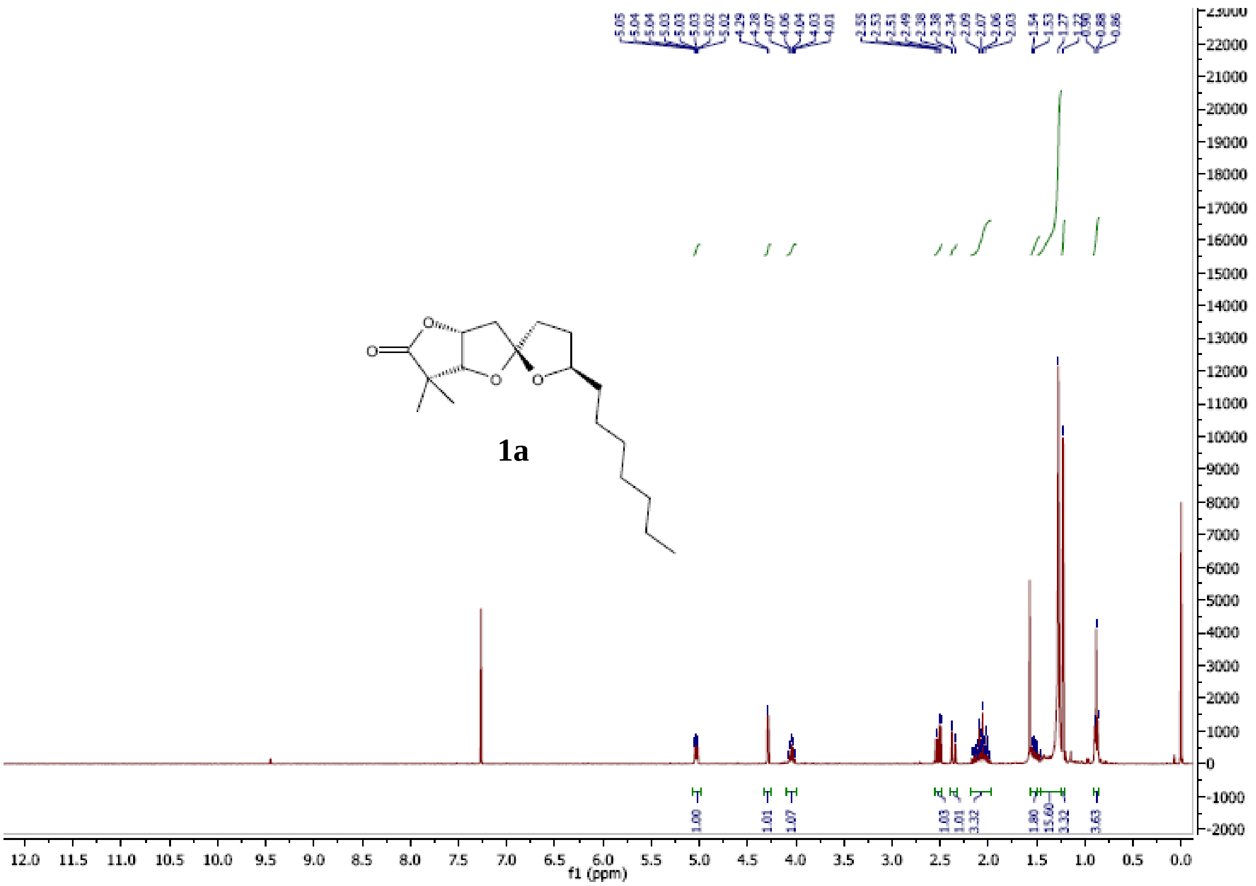
1a
f1 (ppm)

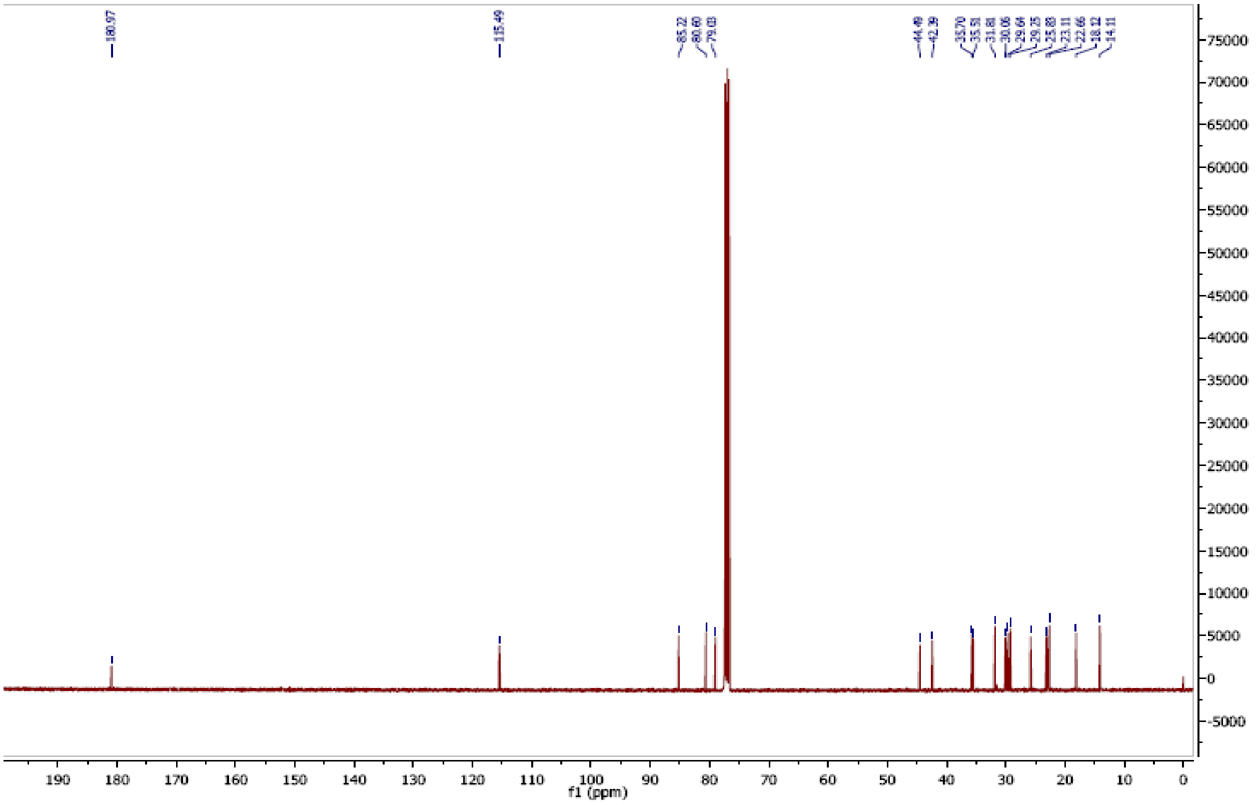
f1 (ppm)

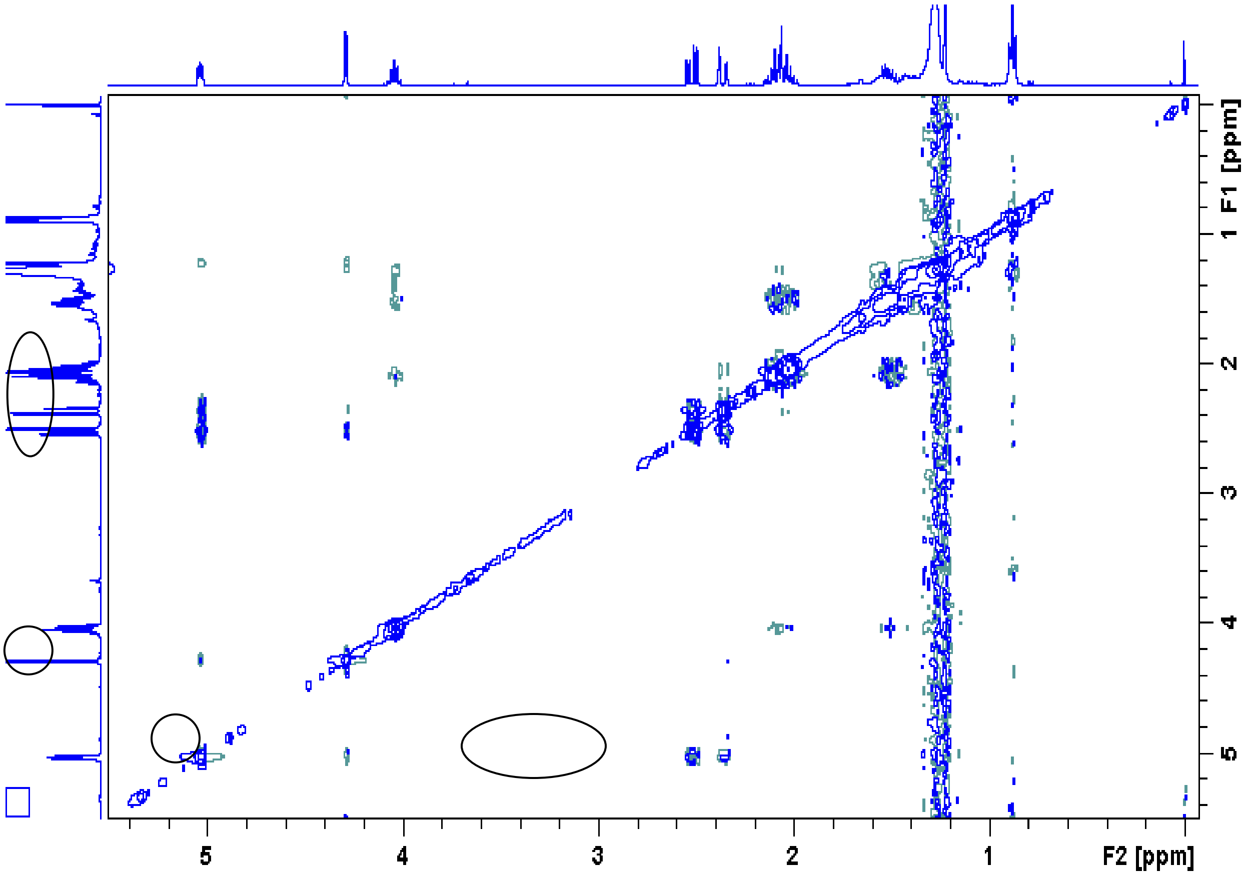
F2 [ppm]
F1 [ppm]
5
4
3
2
1

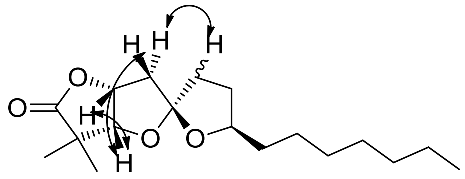
H
H
H
O
O
H
O
O

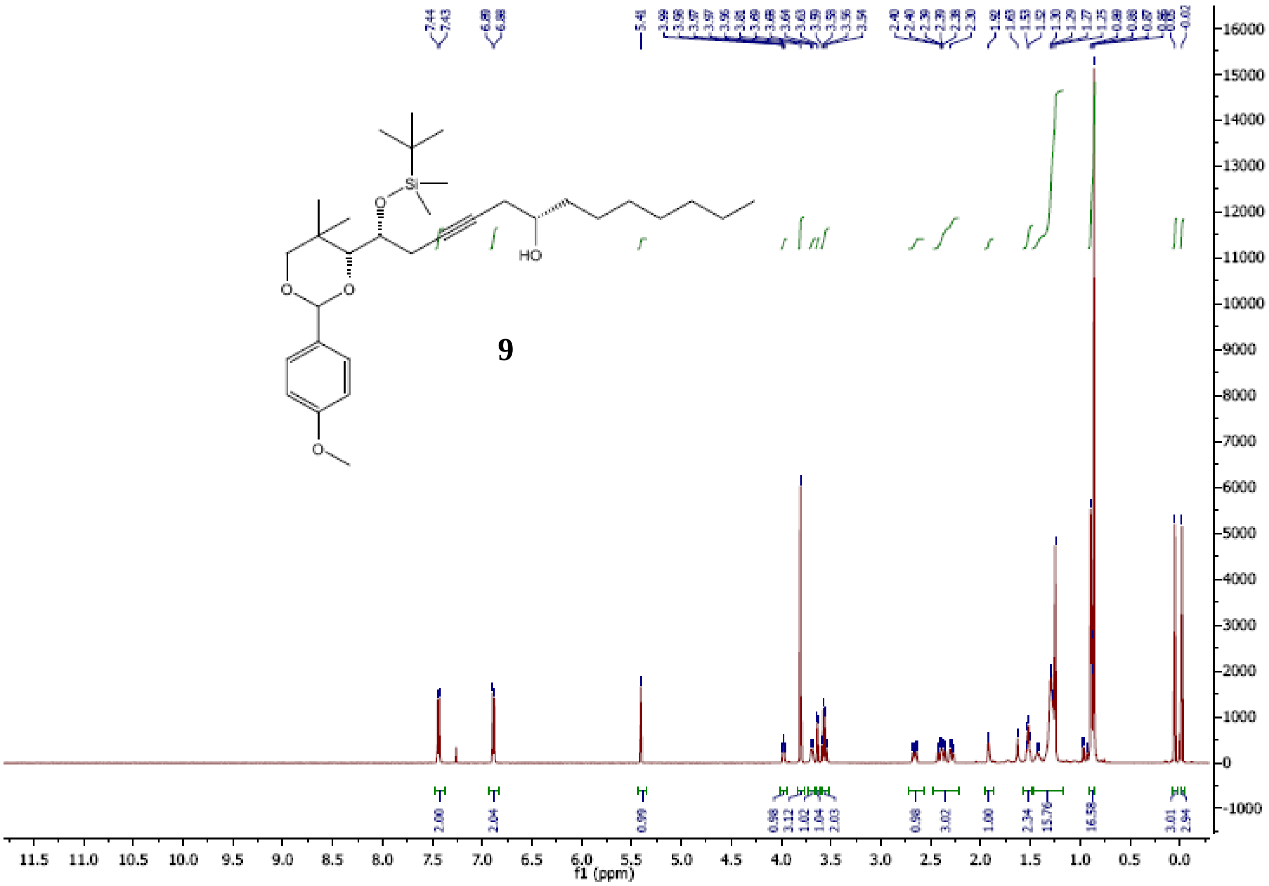
Si
O
O
O
HO
O
9
f1 (ppm)

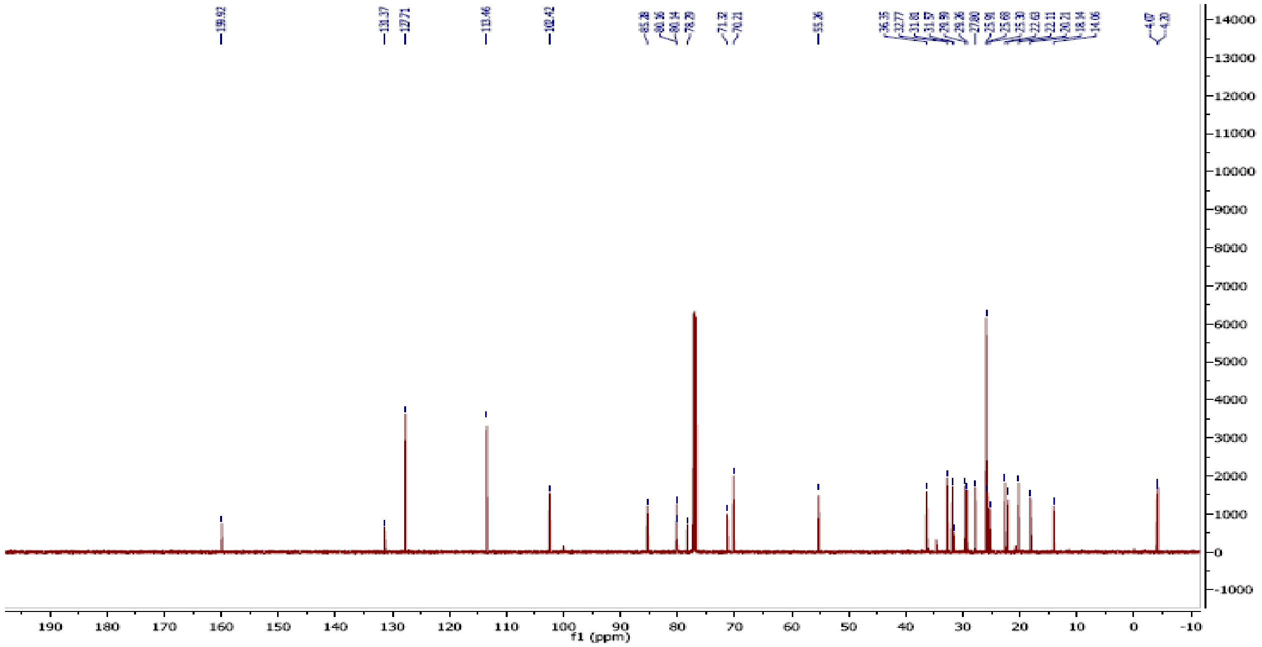
f1 (ppm)

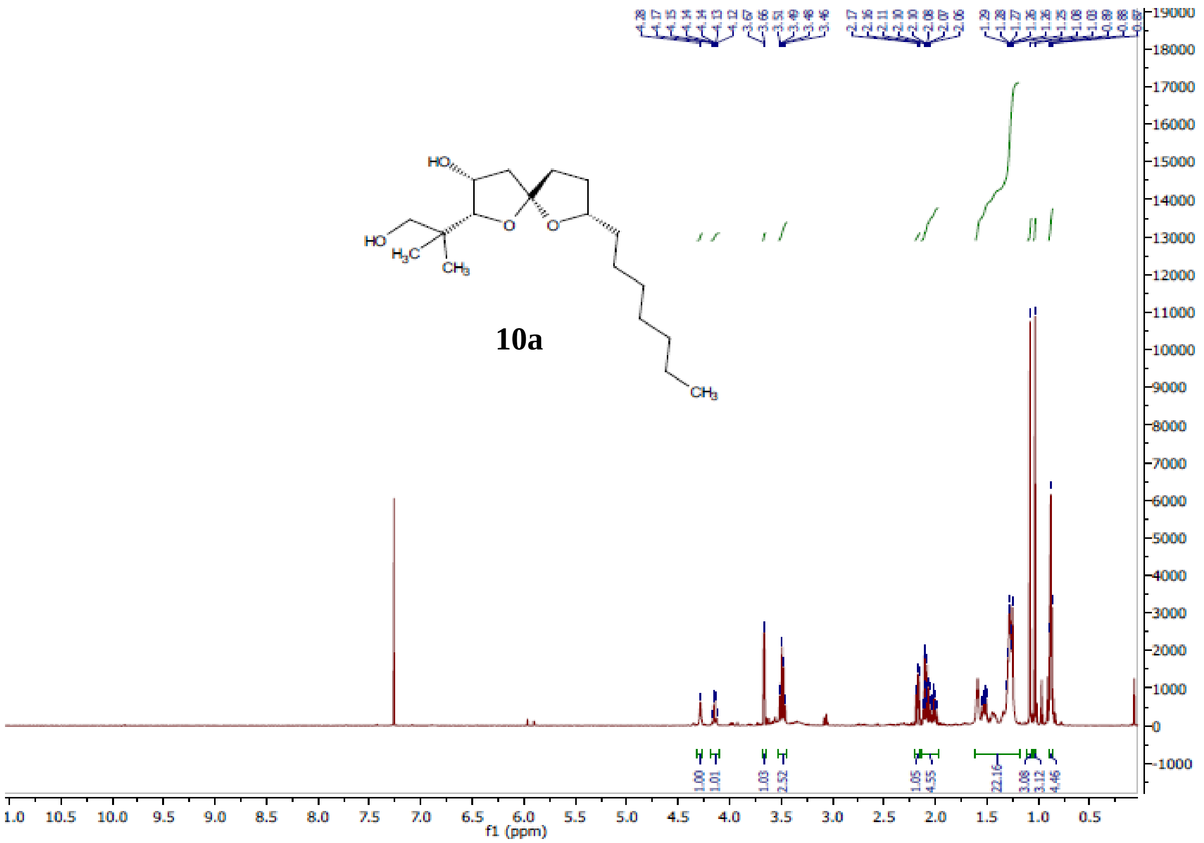
10a
f1 (ppm)

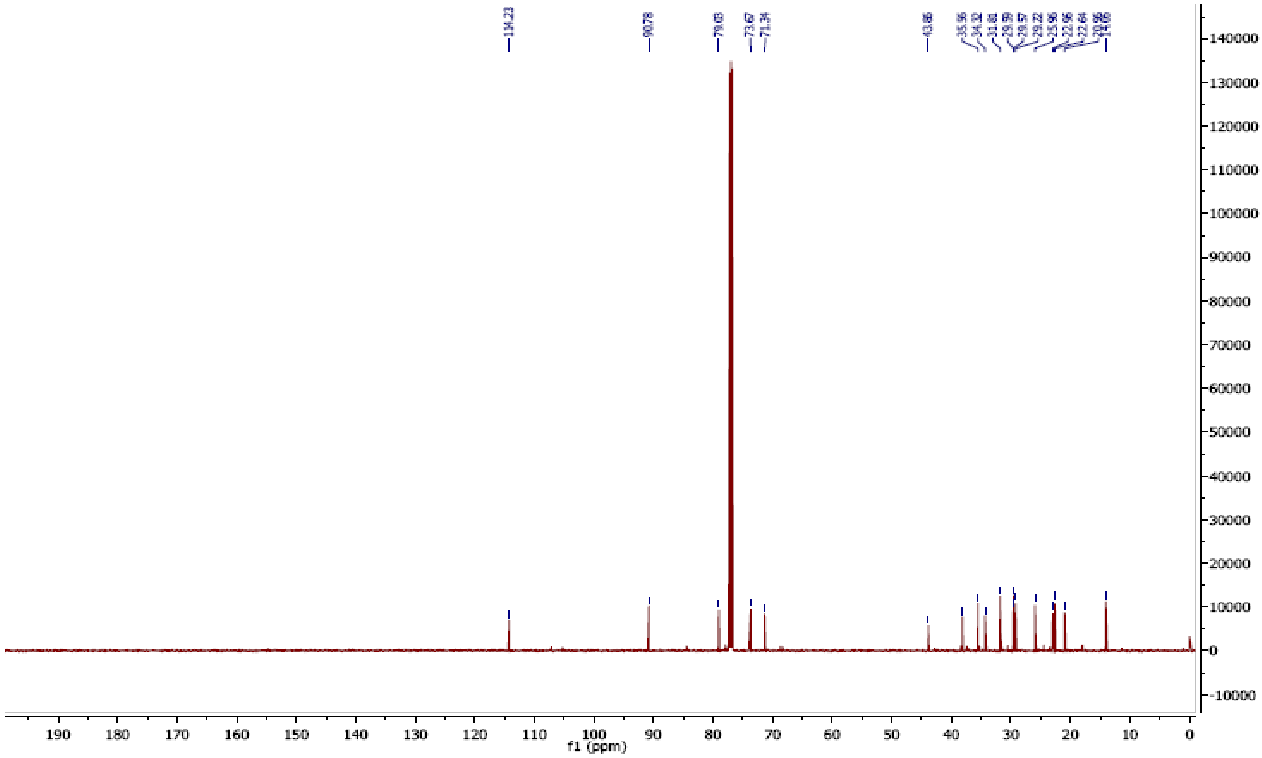
f1 (ppm)

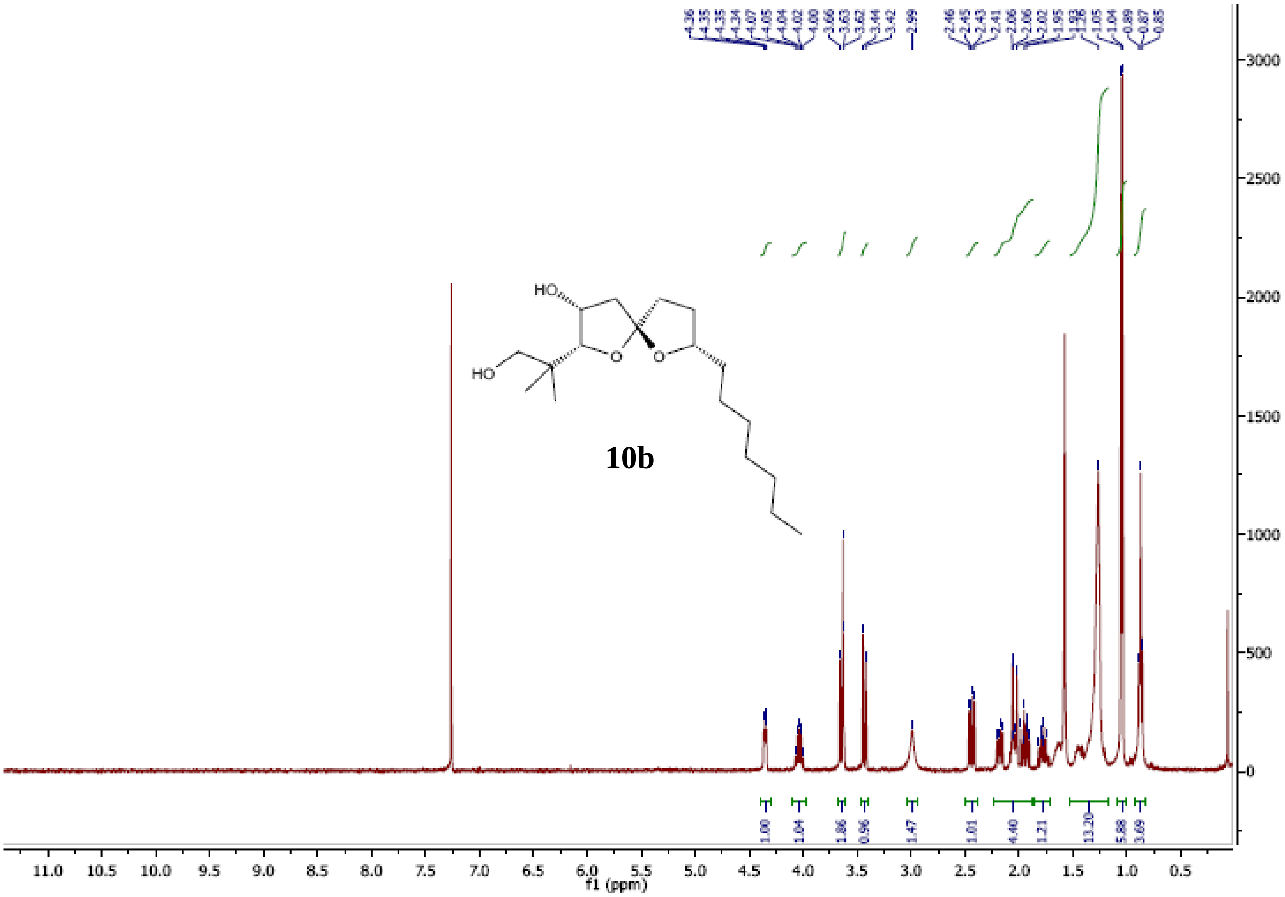
10b
f1 (ppm)

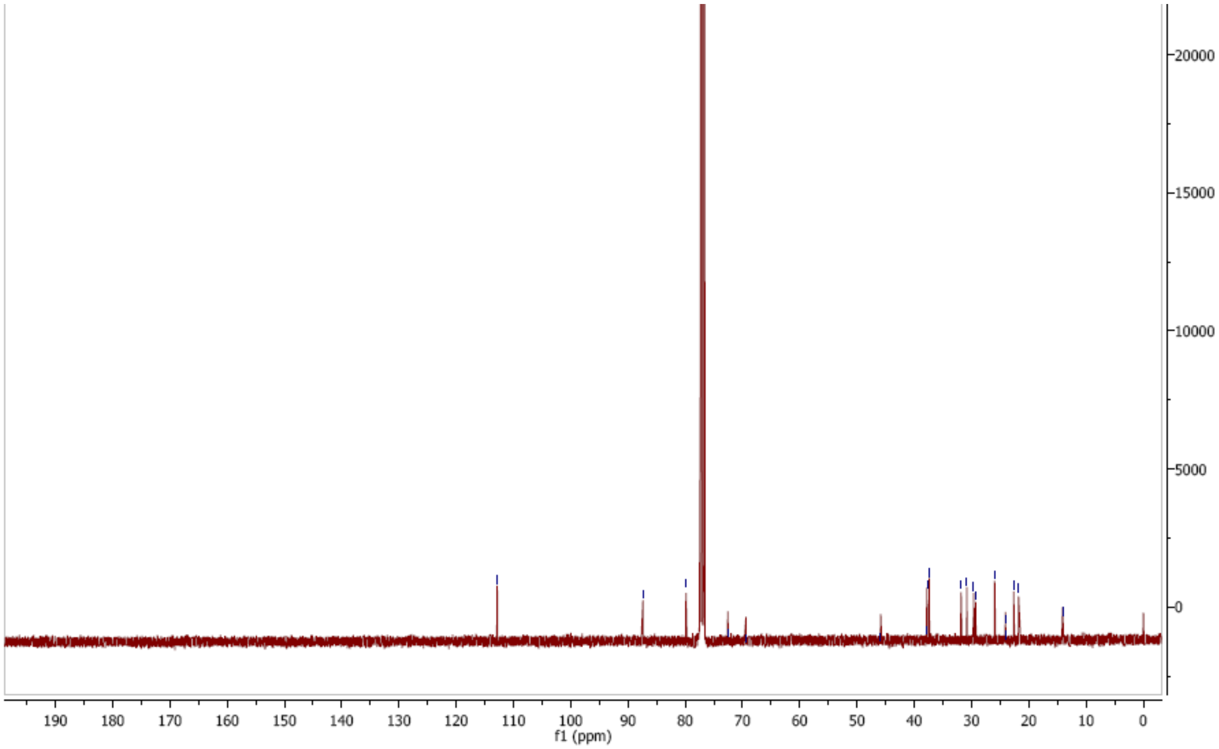
f1 (ppm)

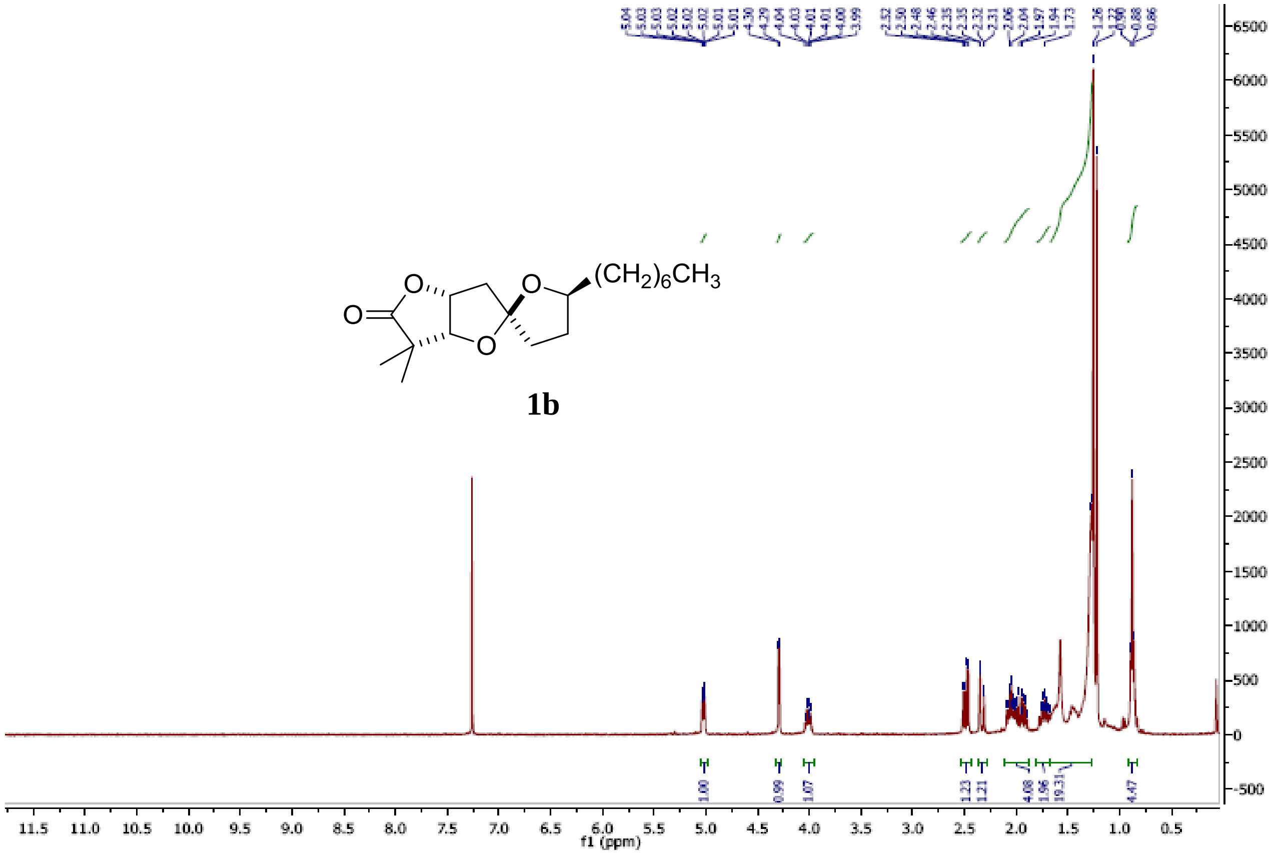

(CH2)6CH3
1.00
0.99
1.07
1.23
1.21
4.08
1.96
19.31
4.47
f1 (ppm)

1b

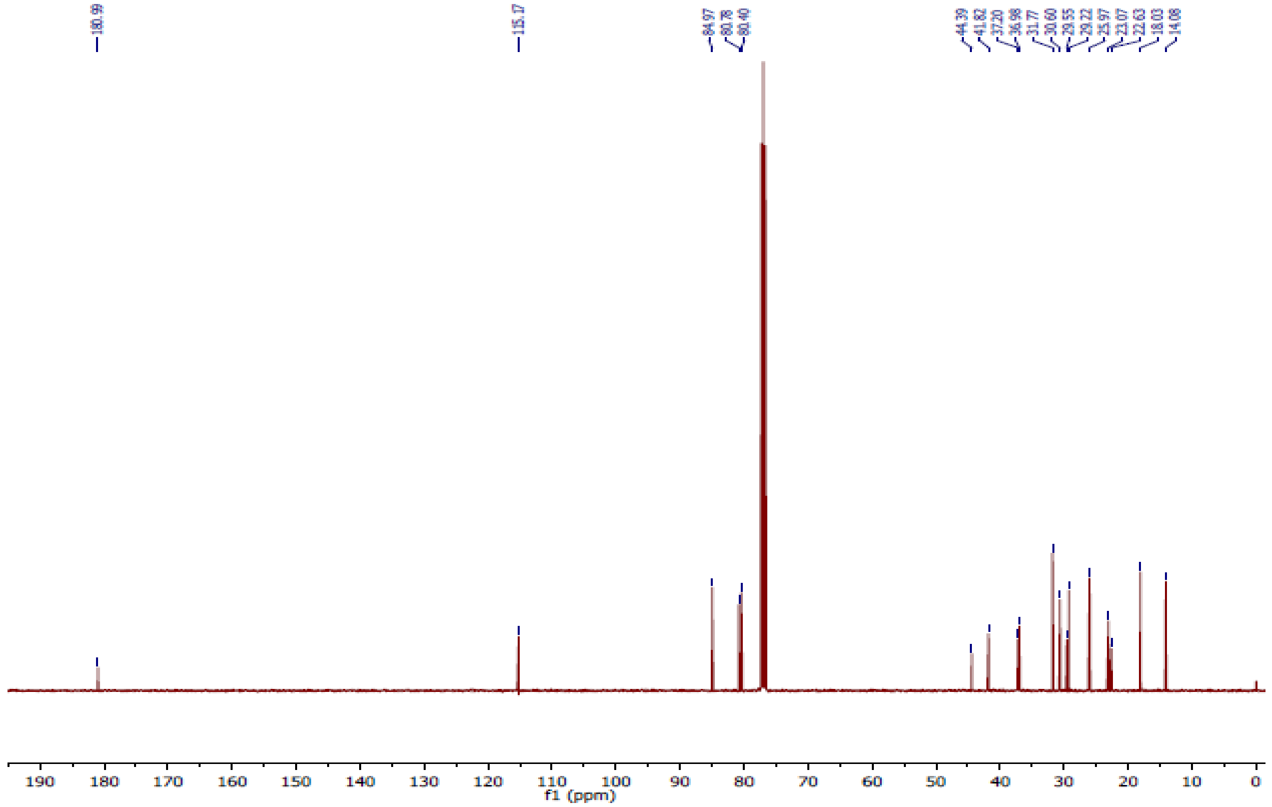

180.99
115.17
84.97
80.78
80.40
44.39
41.82
37.20
36.98
31.77
30.60
29.55
29.22
25.97
23.07
22.63
18.03
14.08
f1 (ppm)

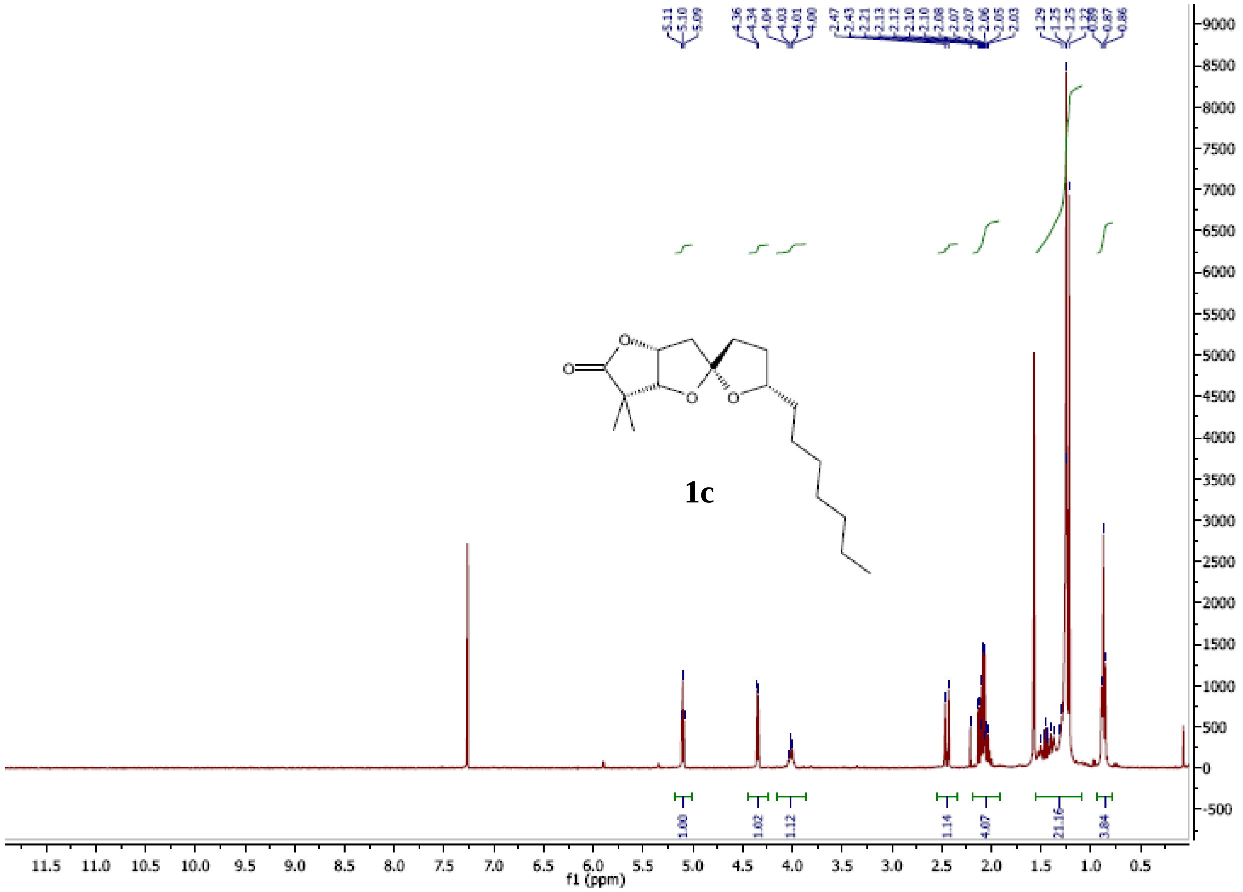
1c
f1 (ppm)

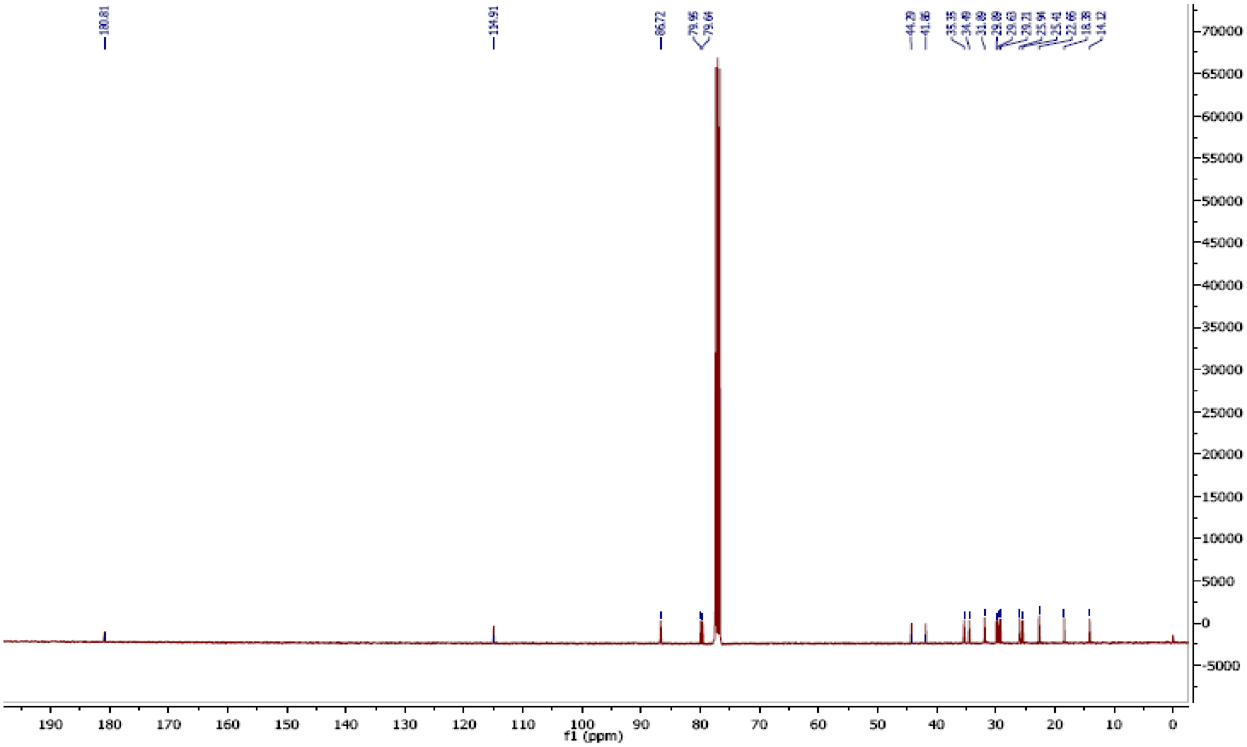
f1 (ppm)

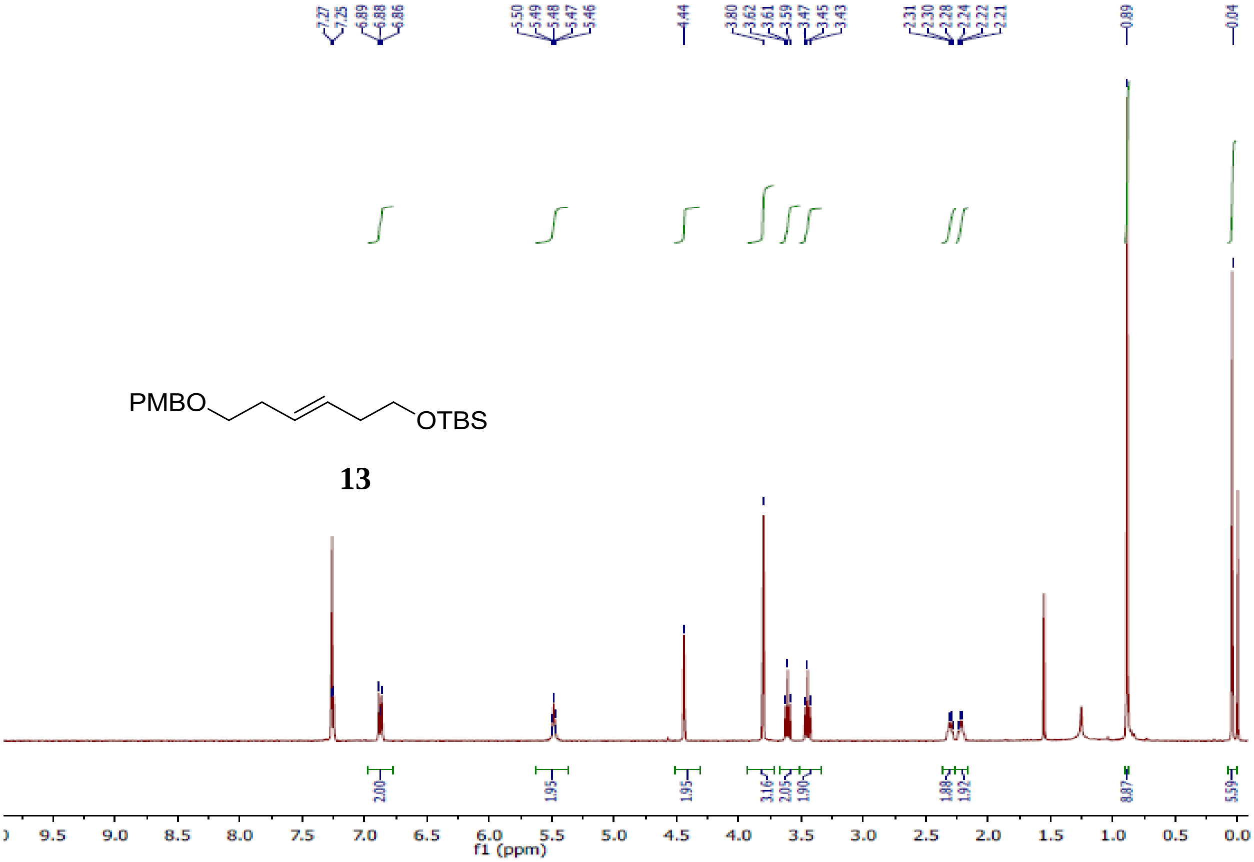
7.27
7.25
6.89
6.88
6.86
5.50
5.49
5.48
5.47
5.46
4.44
3.80
3.62
3.61
3.59
3.47
3.45
3.43
2.31
2.30
2.28
2.24
2.22
2.21
0.89
0.04
PMBO
OTBS
2.00
1.95
1.95
3.16
2.05
1.90
1.88
1.92
8.87
5.59
f1 (ppm)

13

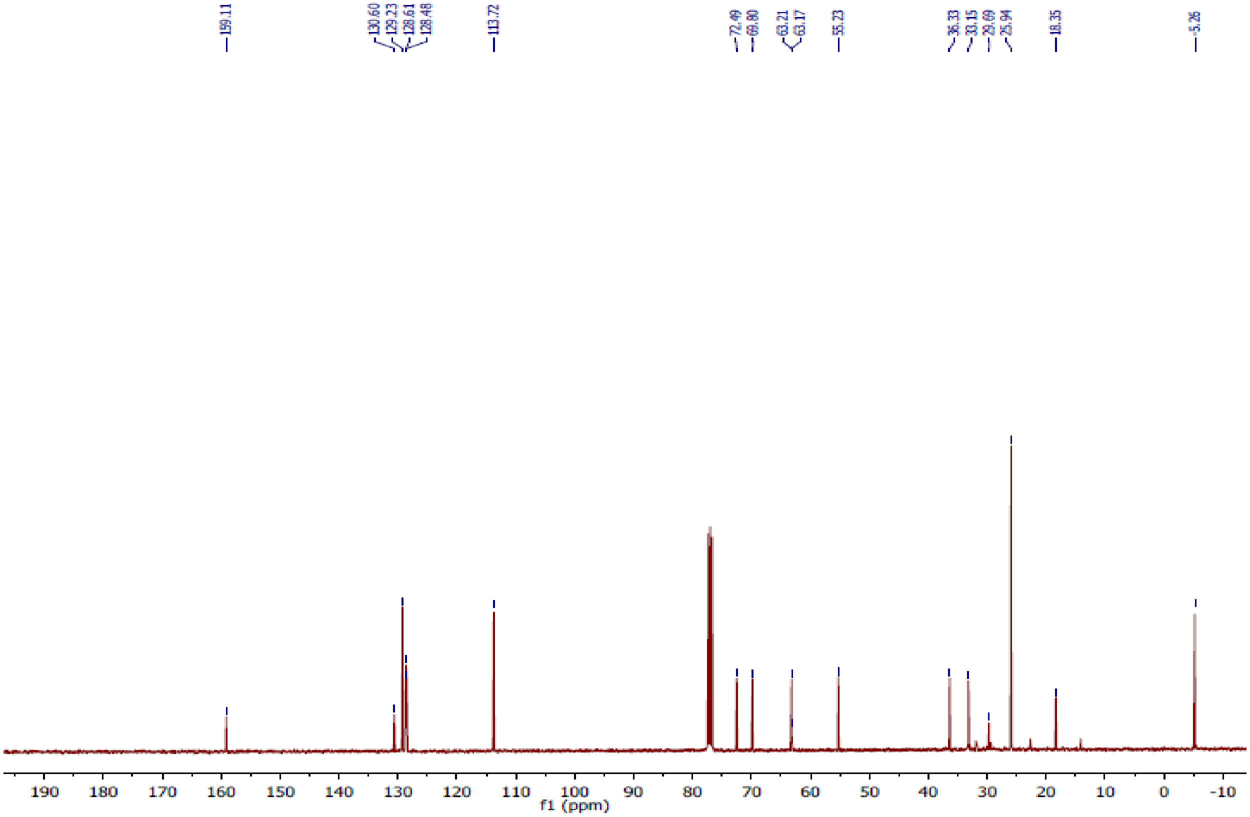
159.11
130.60
129.23
128.61
128.48
113.72
72.49
69.80
63.21
63.17
55.23
36.33
33.15
29.69
25.94
18.35
-5.26
f1 (ppm)

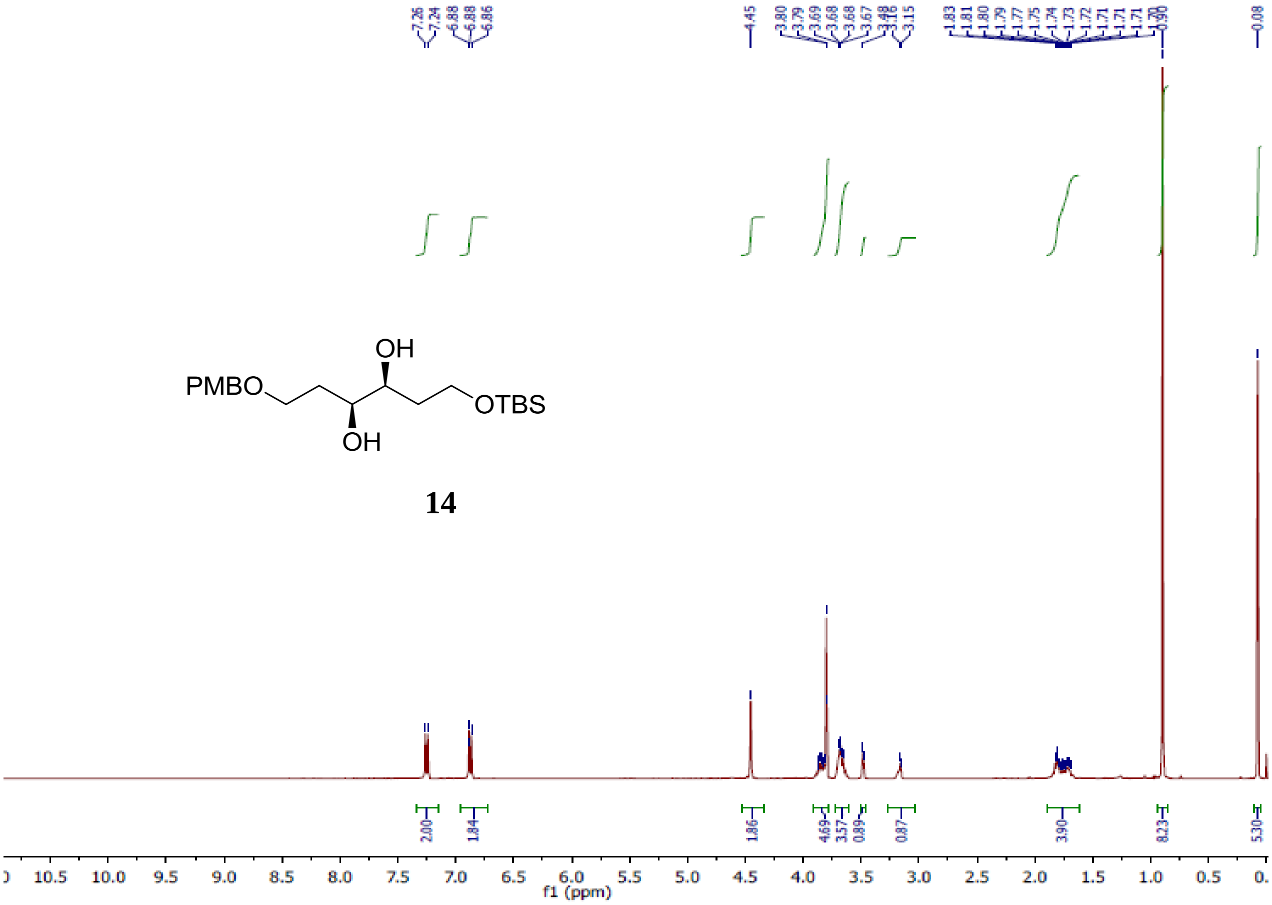

14

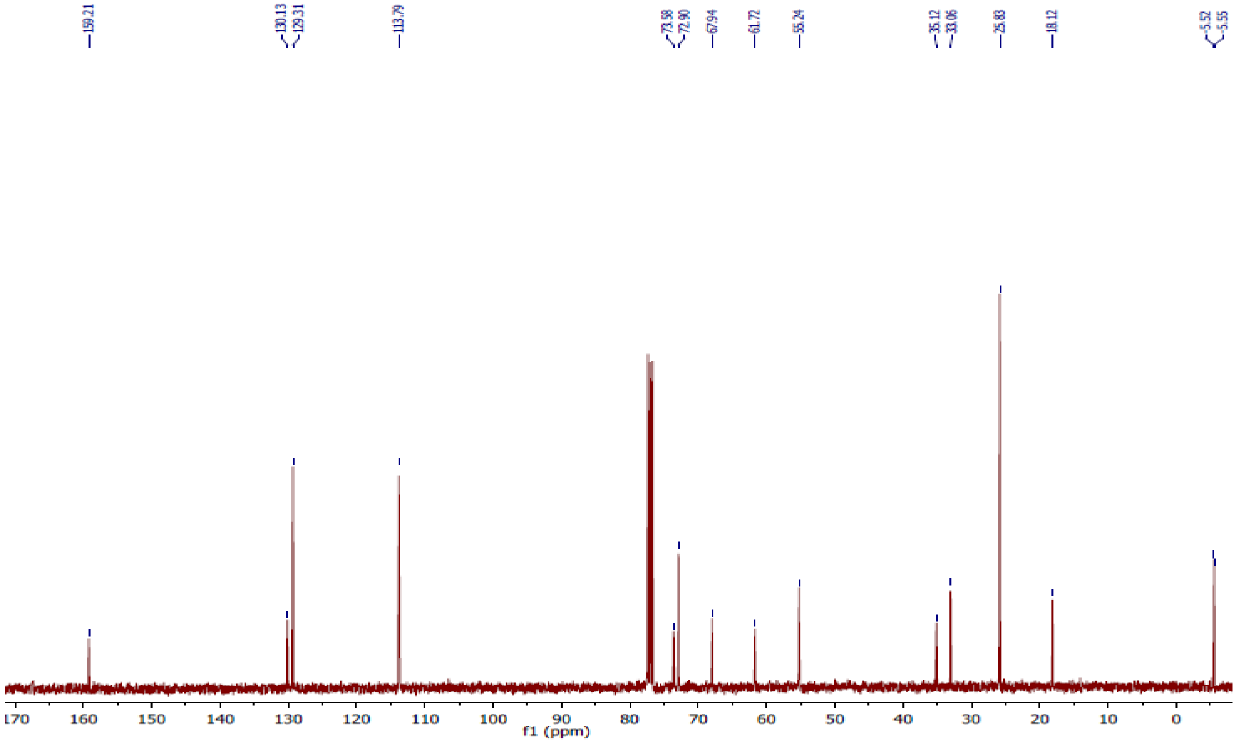

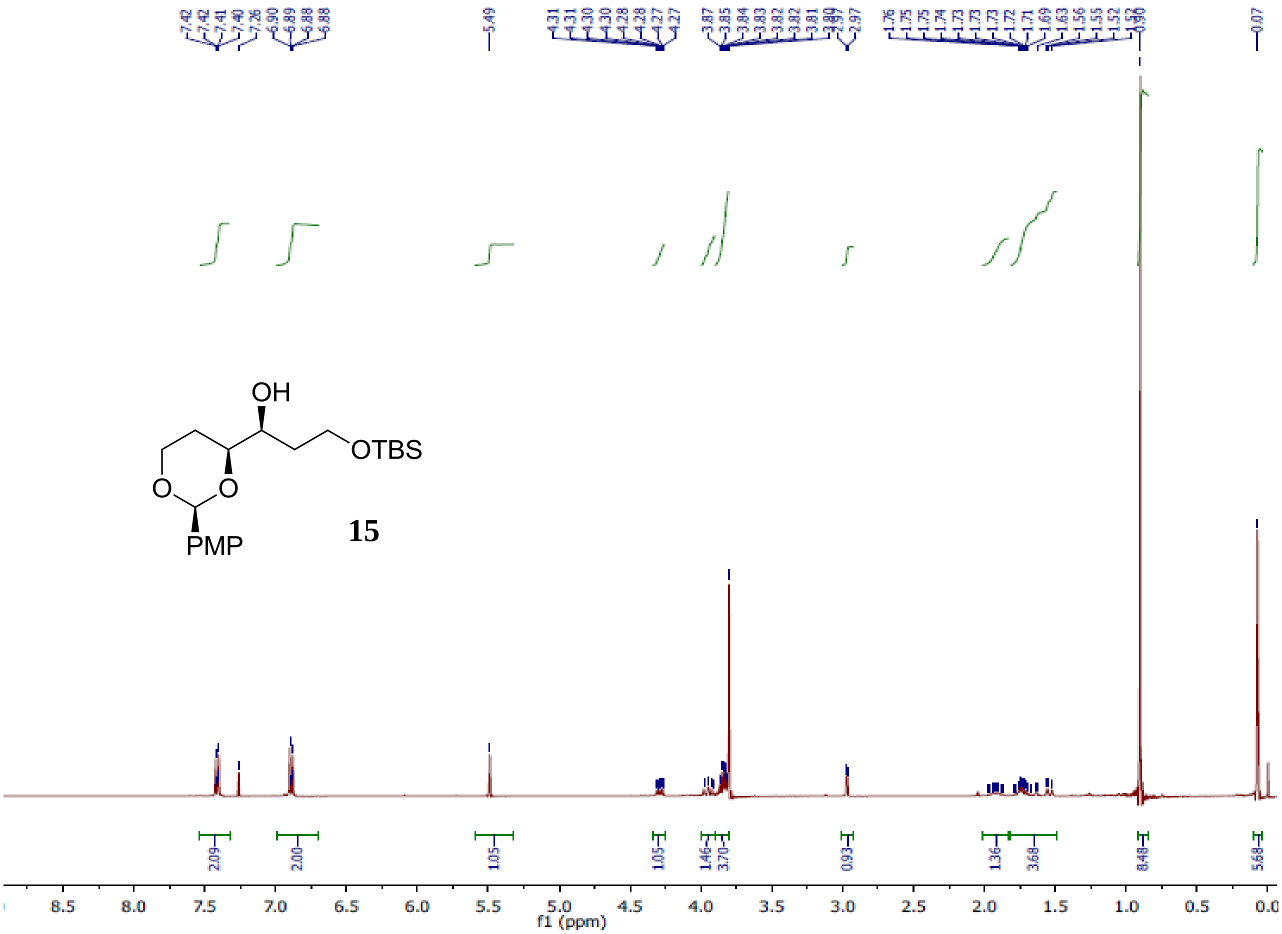
OH
OTBS
O
O
PMP
15
f1 (ppm)

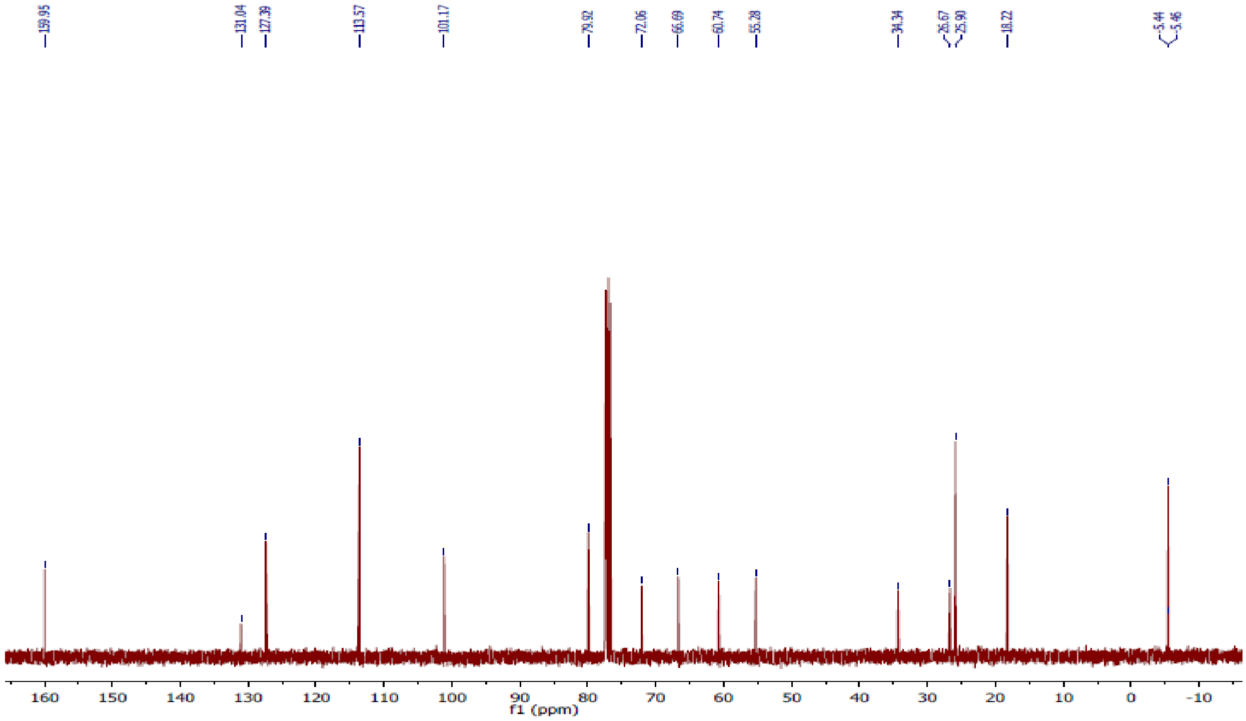
159.95
131.04
127.39
113.57
101.17
79.92
72.06
66.69
60.74
55.28
34.34
26.67
25.90
18.22
-5.44
-5.46
f1 (ppm)

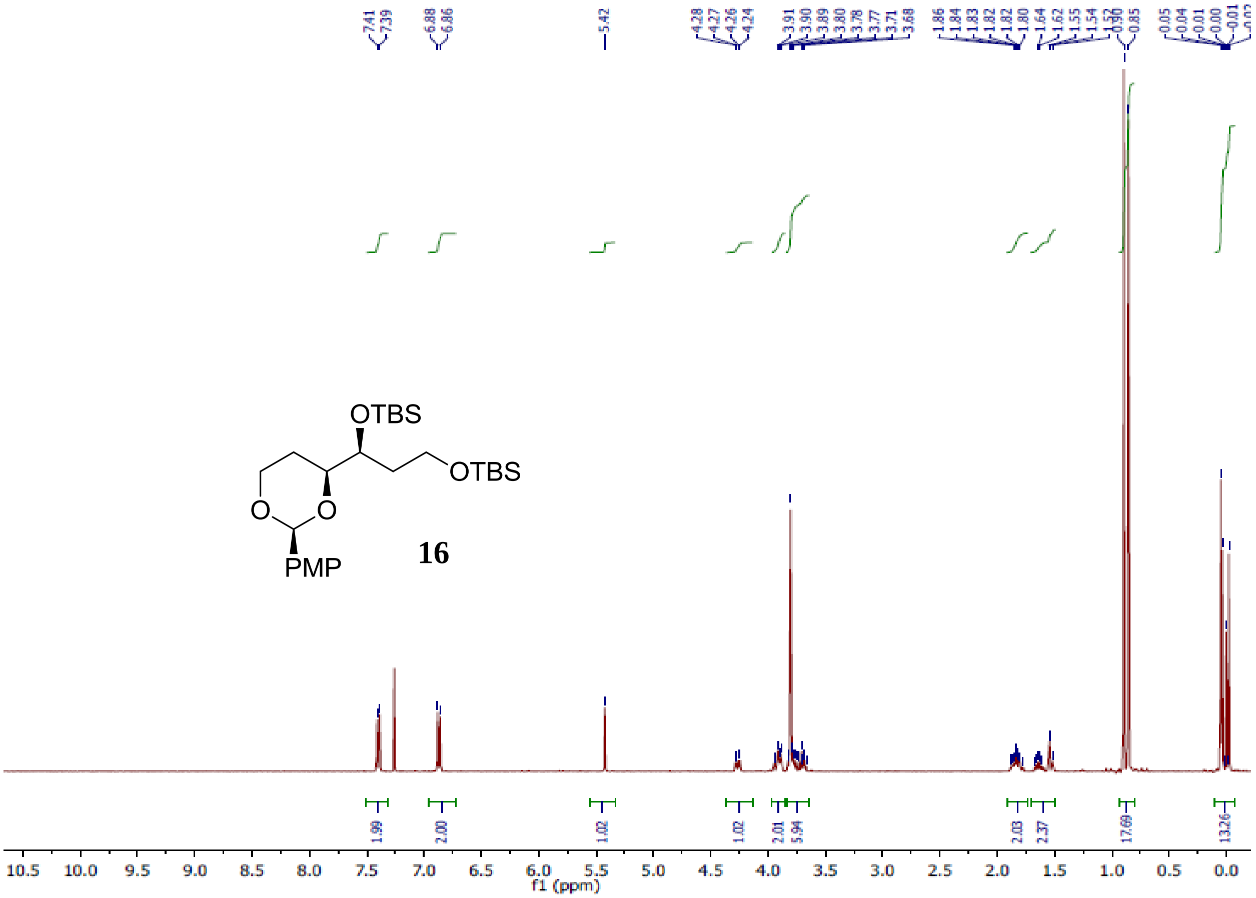
7.41
7.39
6.88
6.86
5.42
4.28
4.27
4.26
4.24
3.91
3.90
3.89
3.80
3.78
3.77
3.71
3.68
1.86
1.84
1.83
1.82
1.82
1.80
1.64
1.62
1.55
1.54
1.52
0.90
0.85
0.05
0.04
0.01
0.00
-0.01
OTBS
OTBS
O
O
PMP
16
1.99
2.00
1.02
1.02
2.01
5.94
2.03
2.37
17.69
13.26
f1 (ppm)

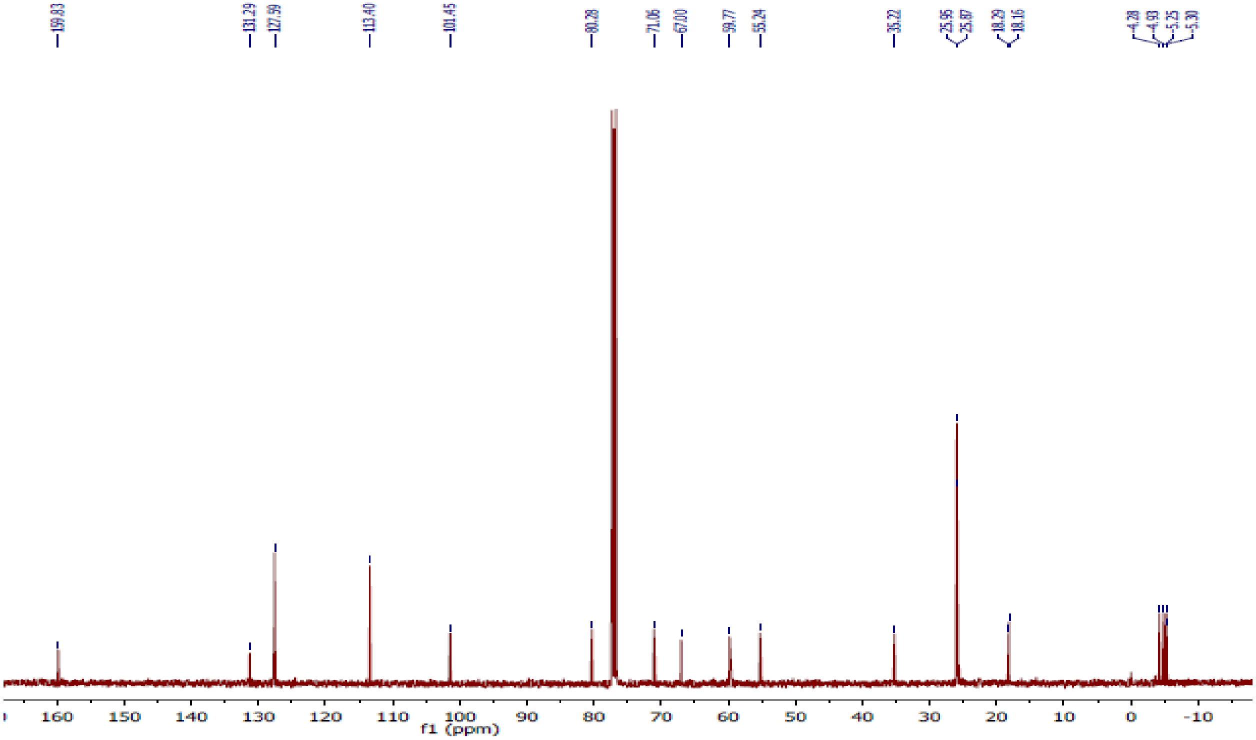
159.83
131.29
127.59
113.40
101.45
80.28
71.06
67.00
59.77
55.24
35.22
25.95
25.87
18.29
18.16
-4.28
-4.93
-5.25
-5.30
f1 (ppm)

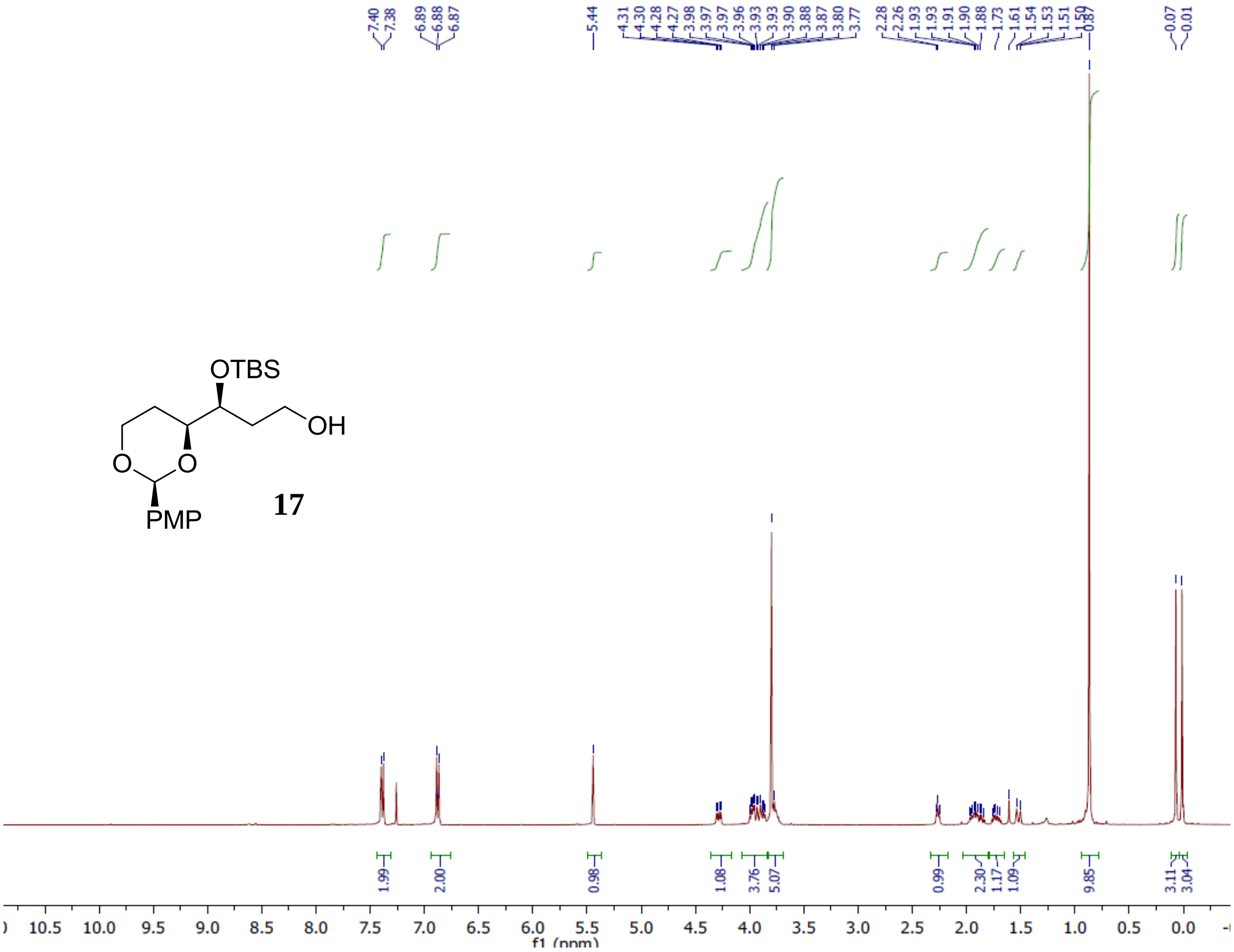
OTBS
OH
O
O
PMP
17
7.40
7.38
6.89
6.88
6.87
5.44
4.31
4.30
4.28
4.27
3.98
3.97
3.97
3.96
3.93
3.93
3.90
3.88
3.87
3.80
3.77
2.28
2.26
1.93
1.93
1.91
1.90
1.88
1.73
1.61
1.54
1.53
1.51
0.87
0.07
0.01
1.99
2.00
0.98
1.08
3.76
5.07
0.99
2.30
1.17
1.09
9.85
3.11
3.04
10.5 10.0 9.5 9.0 8.5 8.0 7.5 7.0 6.5 6.0 5.5 5.0 4.5 4.0 3.5 3.0 2.5 2.0 1.5 1.0 0.5 0.0
f1 (ppm)

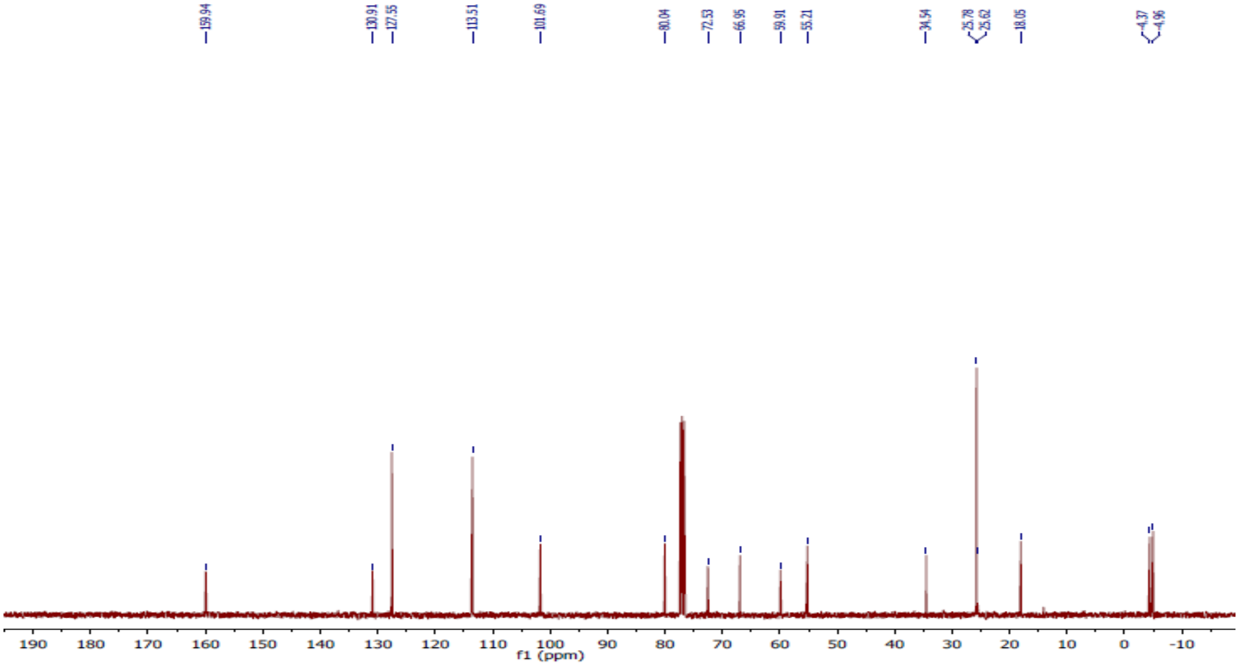
159.94
130.91
127.55
113.51
101.69
80.04
72.53
66.95
59.91
55.21
34.54
25.78
25.62
18.05
-4.37
-4.96
190 180 170 160 150 140 130 120 110 100 90 80 70 60 50 40 30 20 10 0 -10
f1 (ppm)

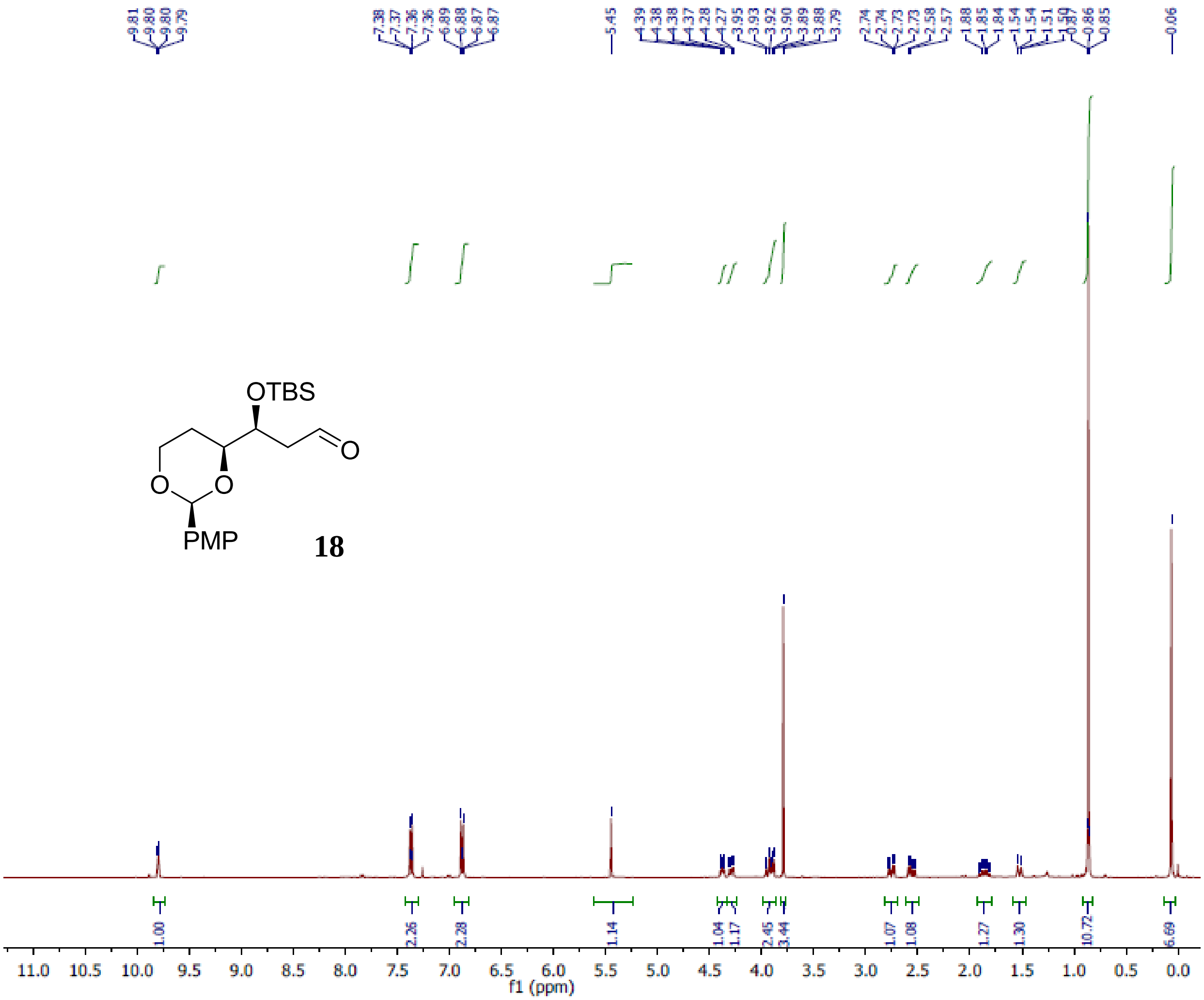
OTBS
O
O
O
PMP
18
f1 (ppm)

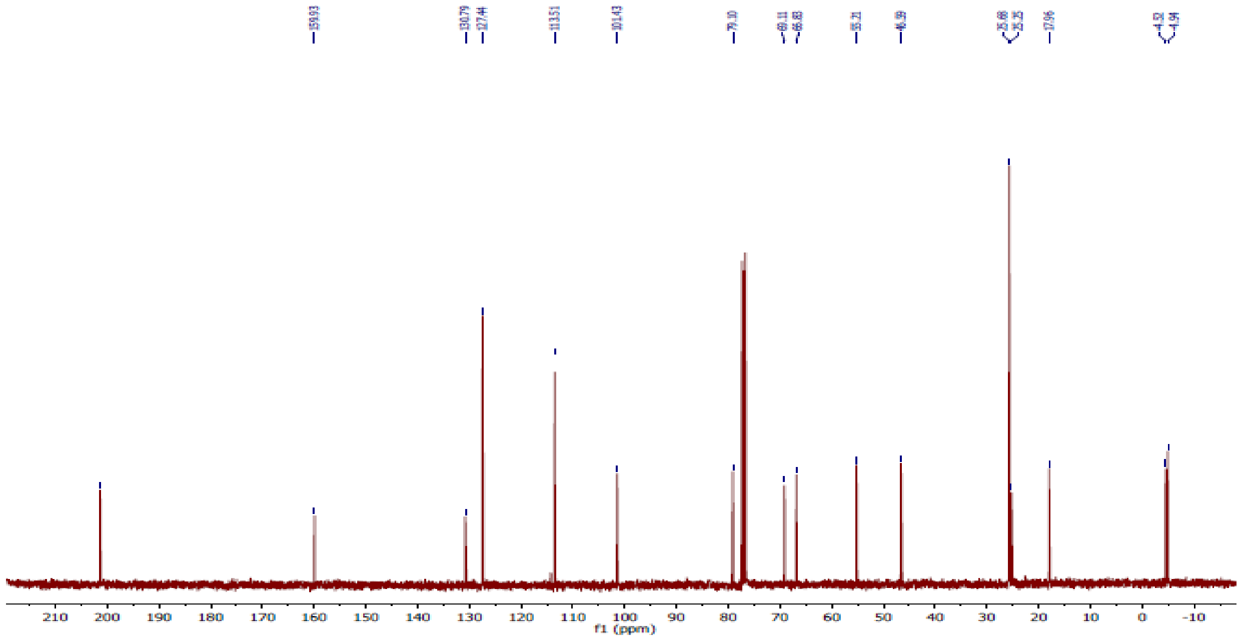
f1 (ppm)

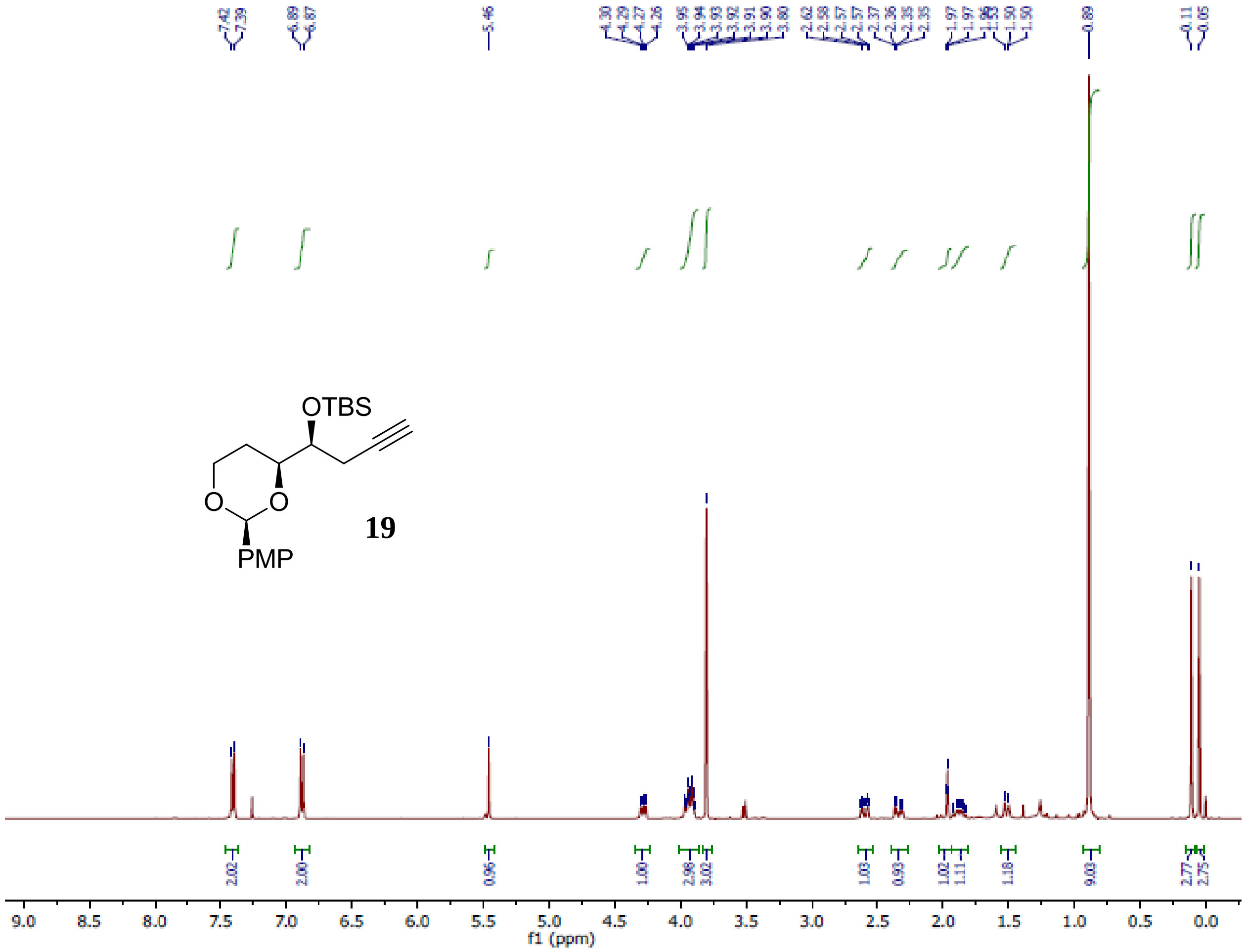

19

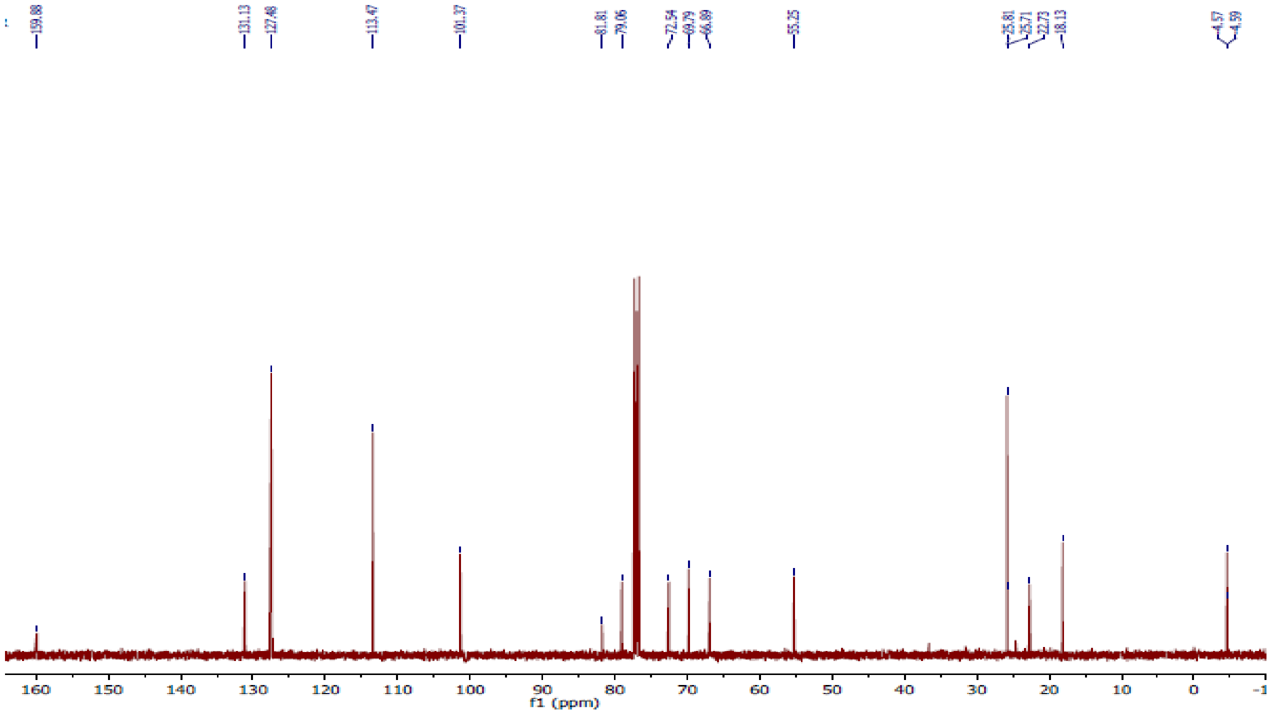

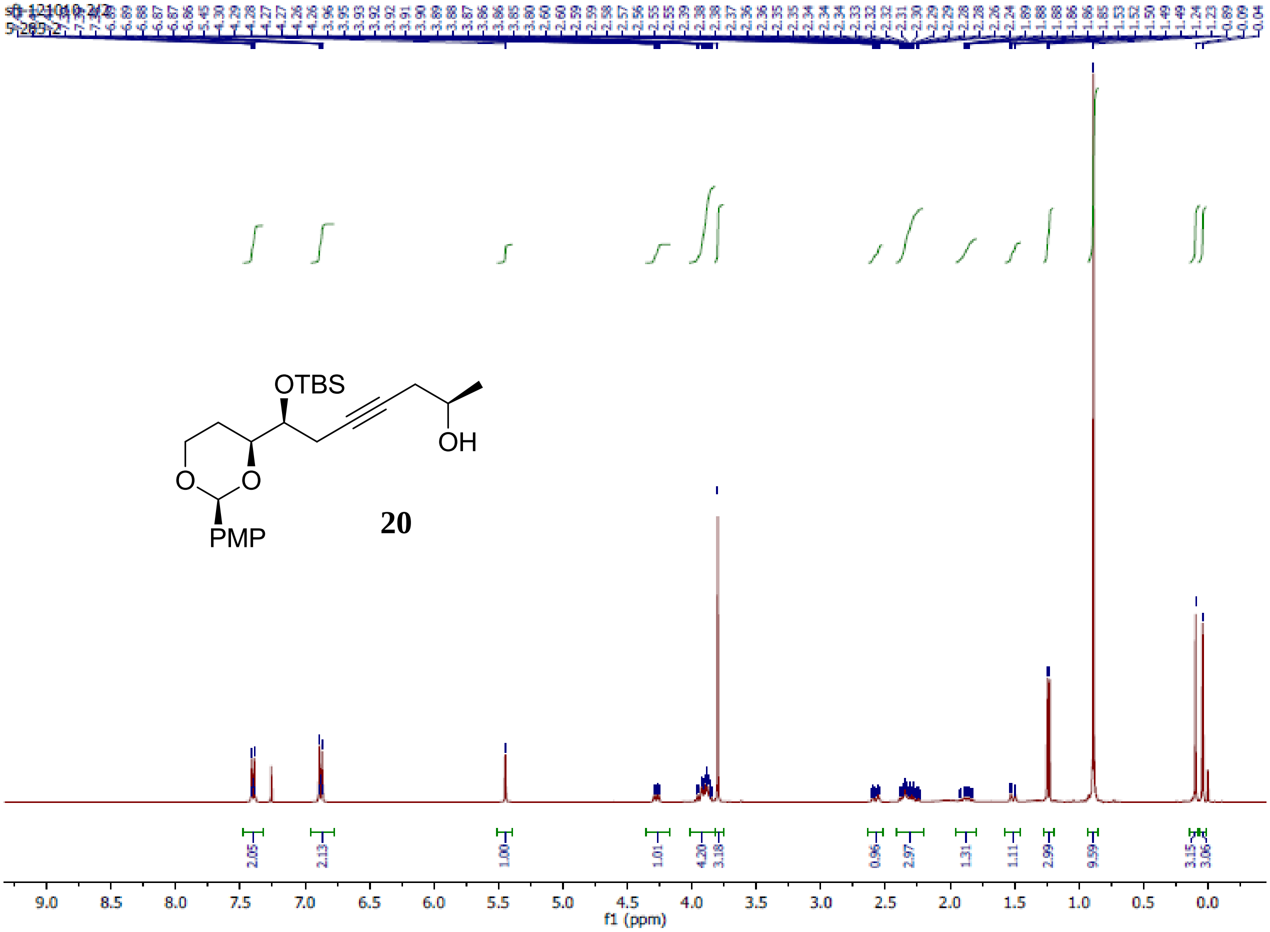
OTBS
OH
O
O
PMP
20
f1 (ppm)

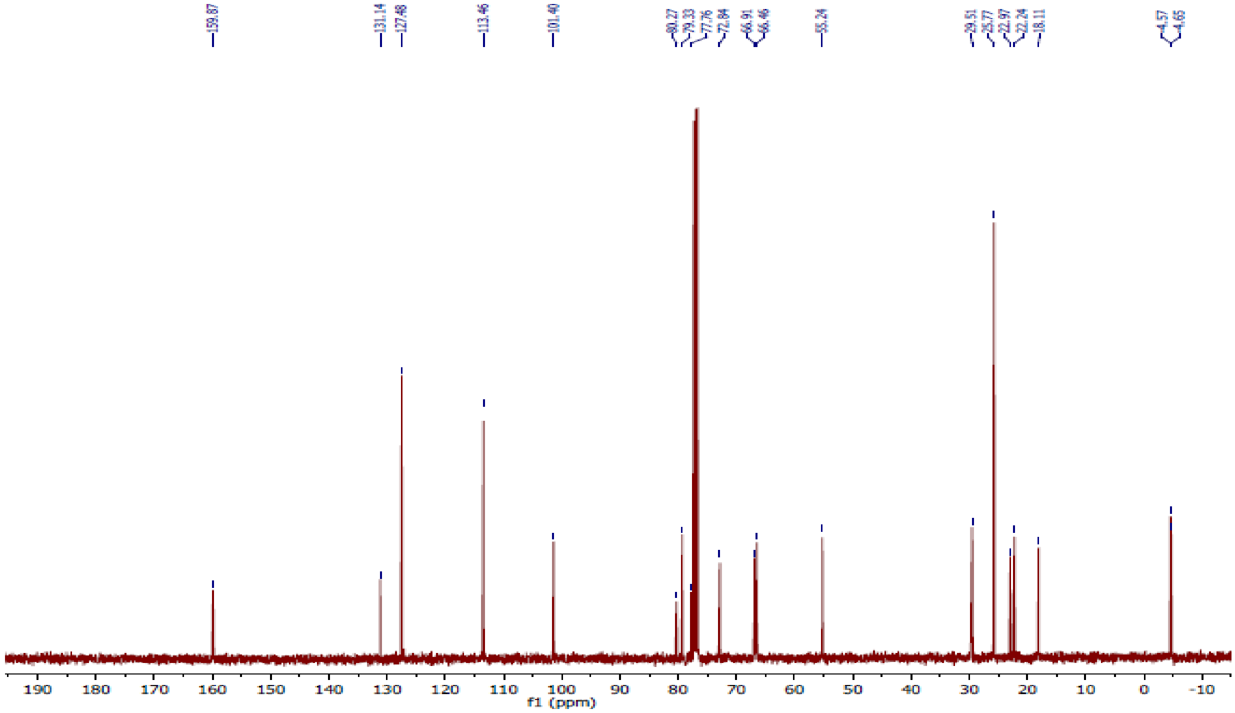
f1 (ppm)

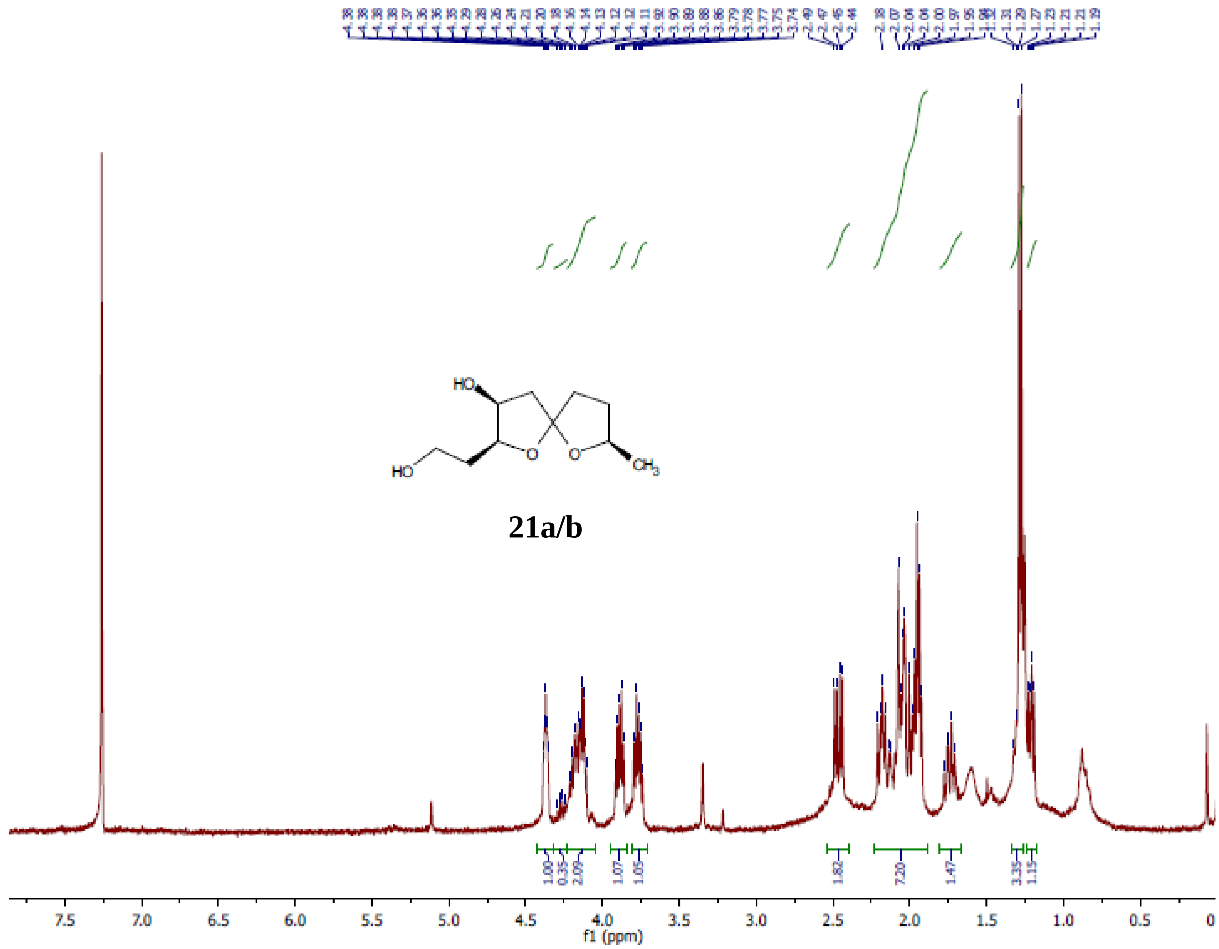

21a/b

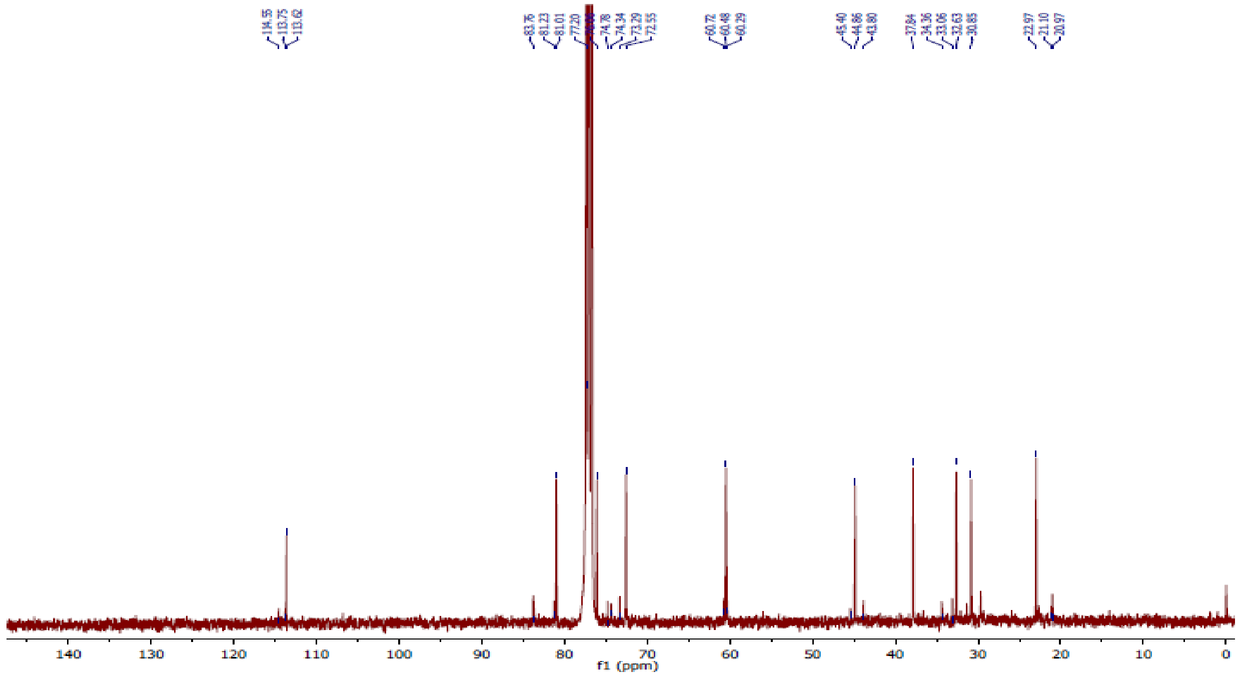

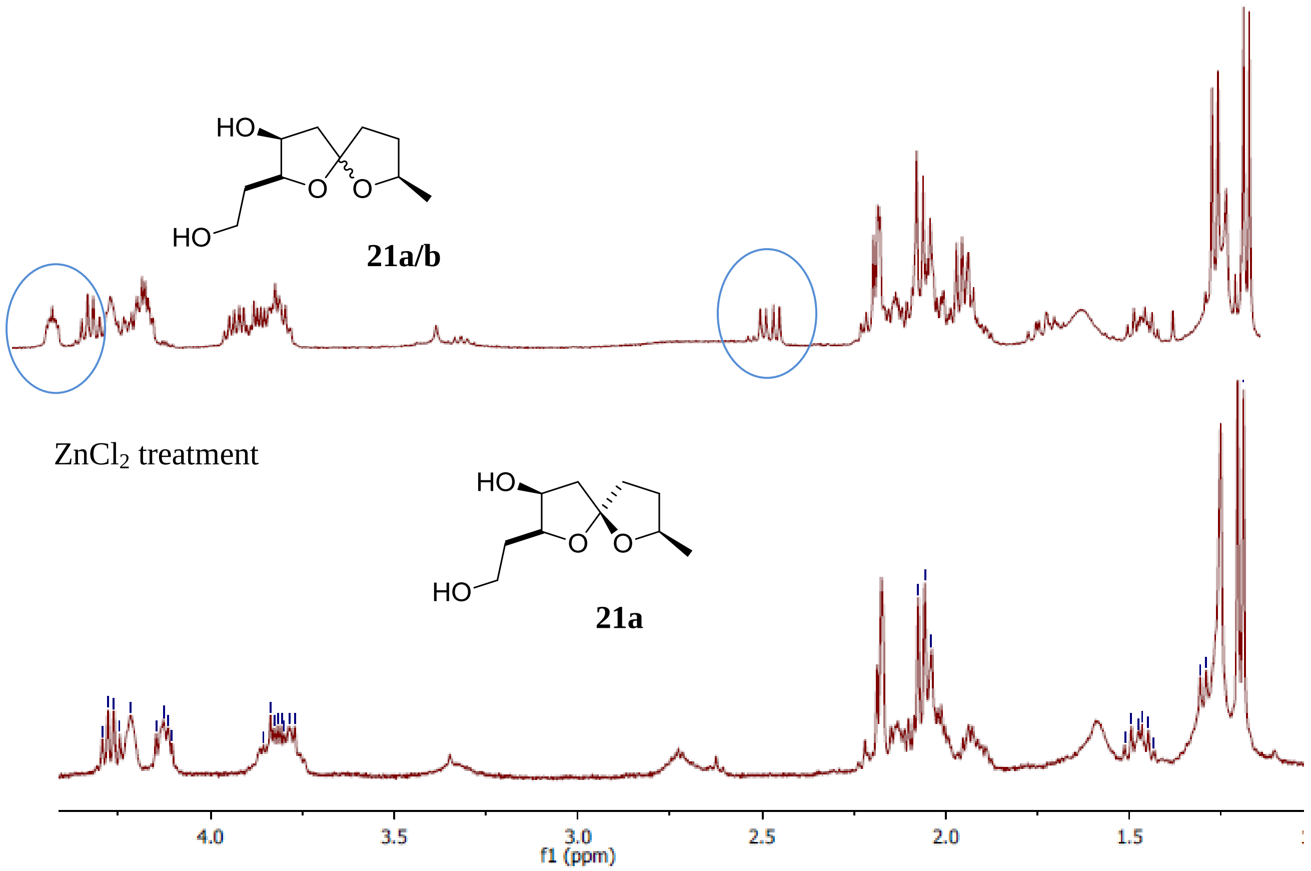
HO
HO
O
O
21a/b
ZnCl$_2$ treatment
HO
HO
O
O
21a
4.0
3.5
3.0
2.5
2.0
1.5
f1 (ppm)

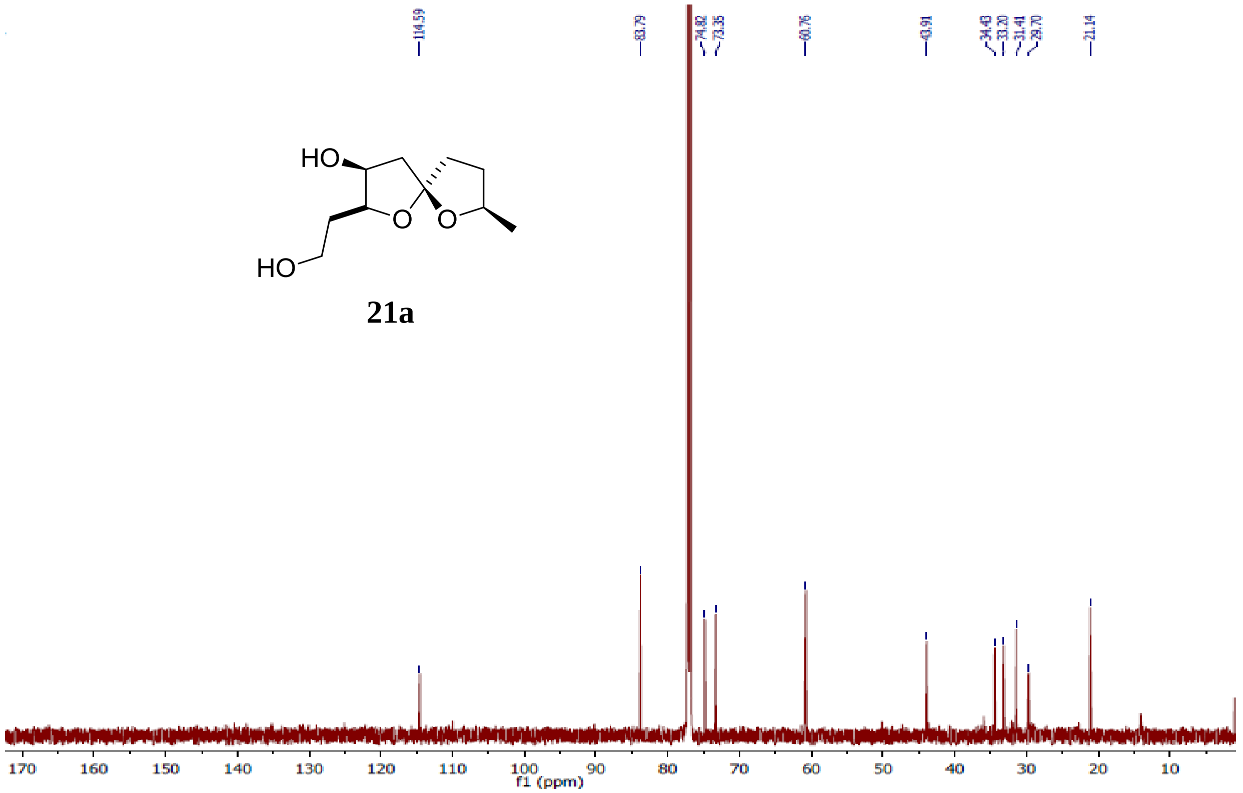
114.59
83.79
74.82
73.35
60.76
43.91
34.43
33.20
31.41
29.70
21.14
HO
HO
O
O
21a
170
160
150
140
130
120
110
100
90
80
70
60
50
40
30
20
10
f1 (ppm)

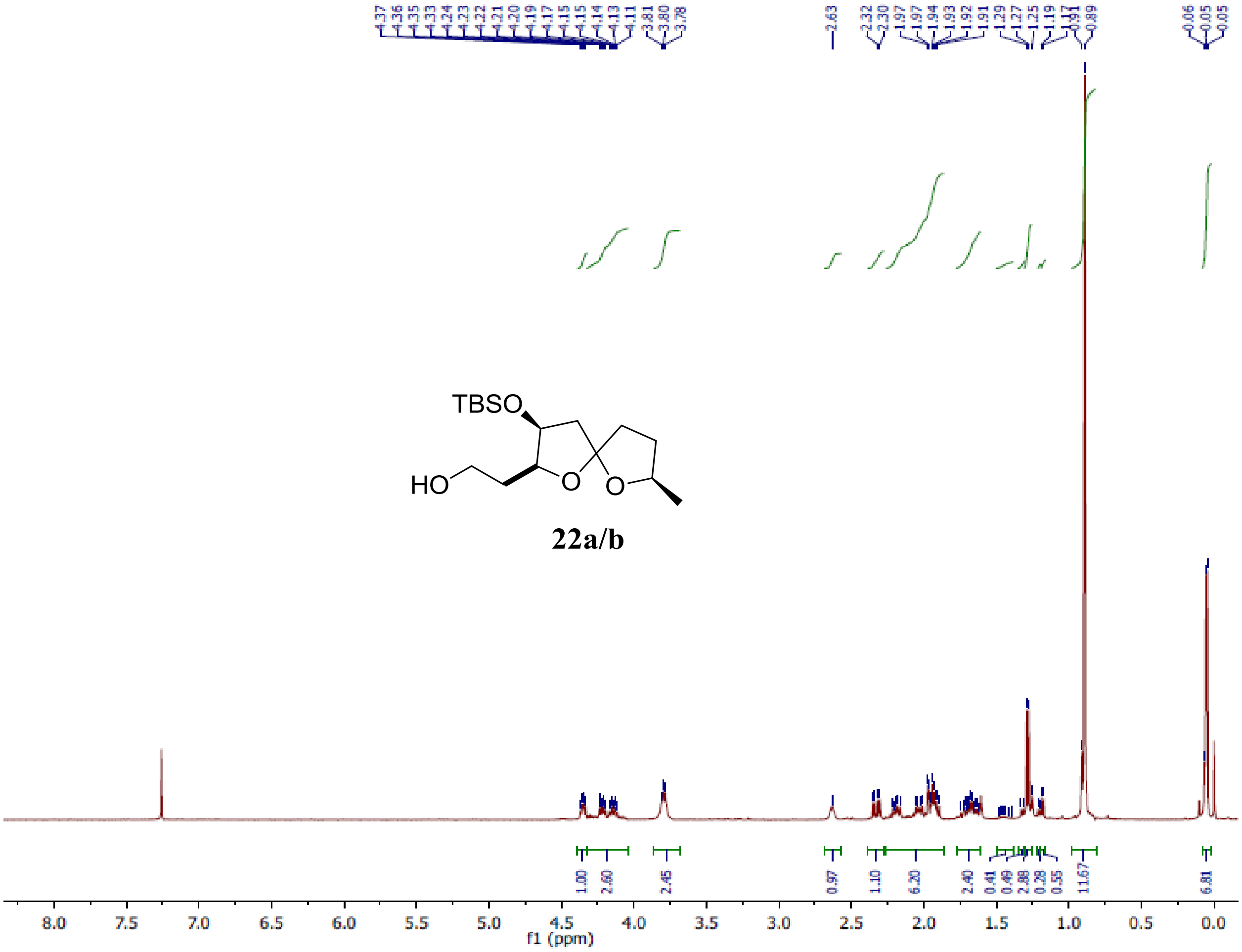

22a/b

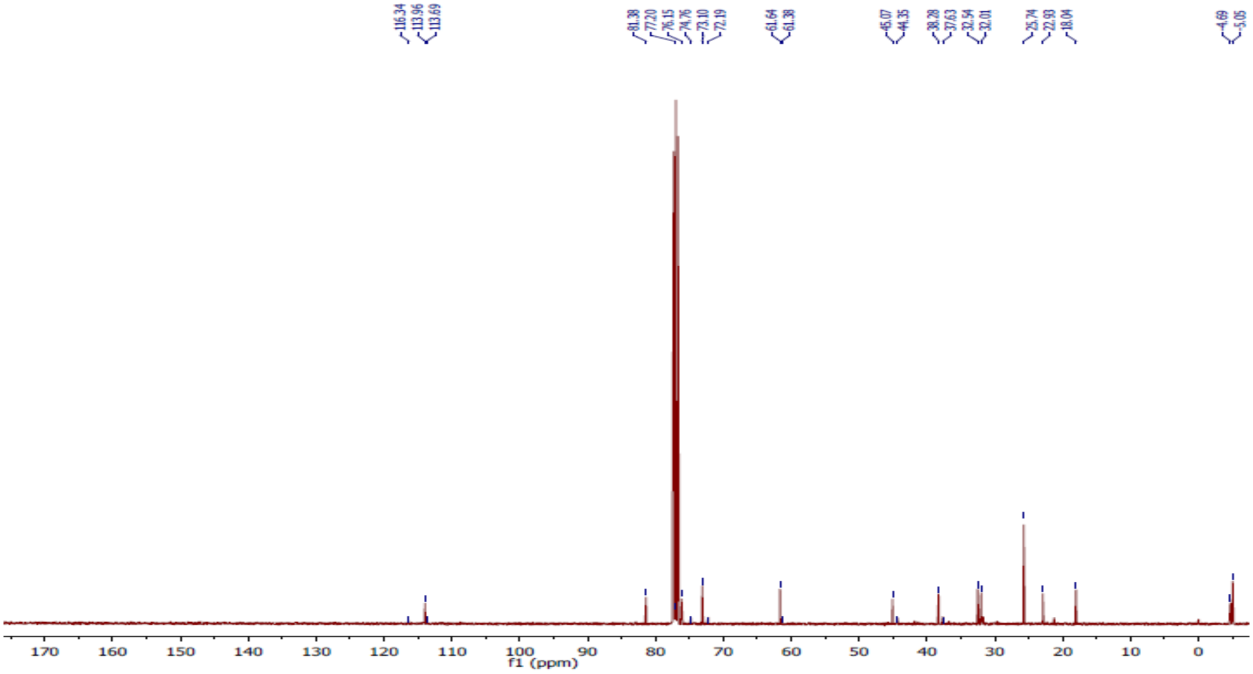

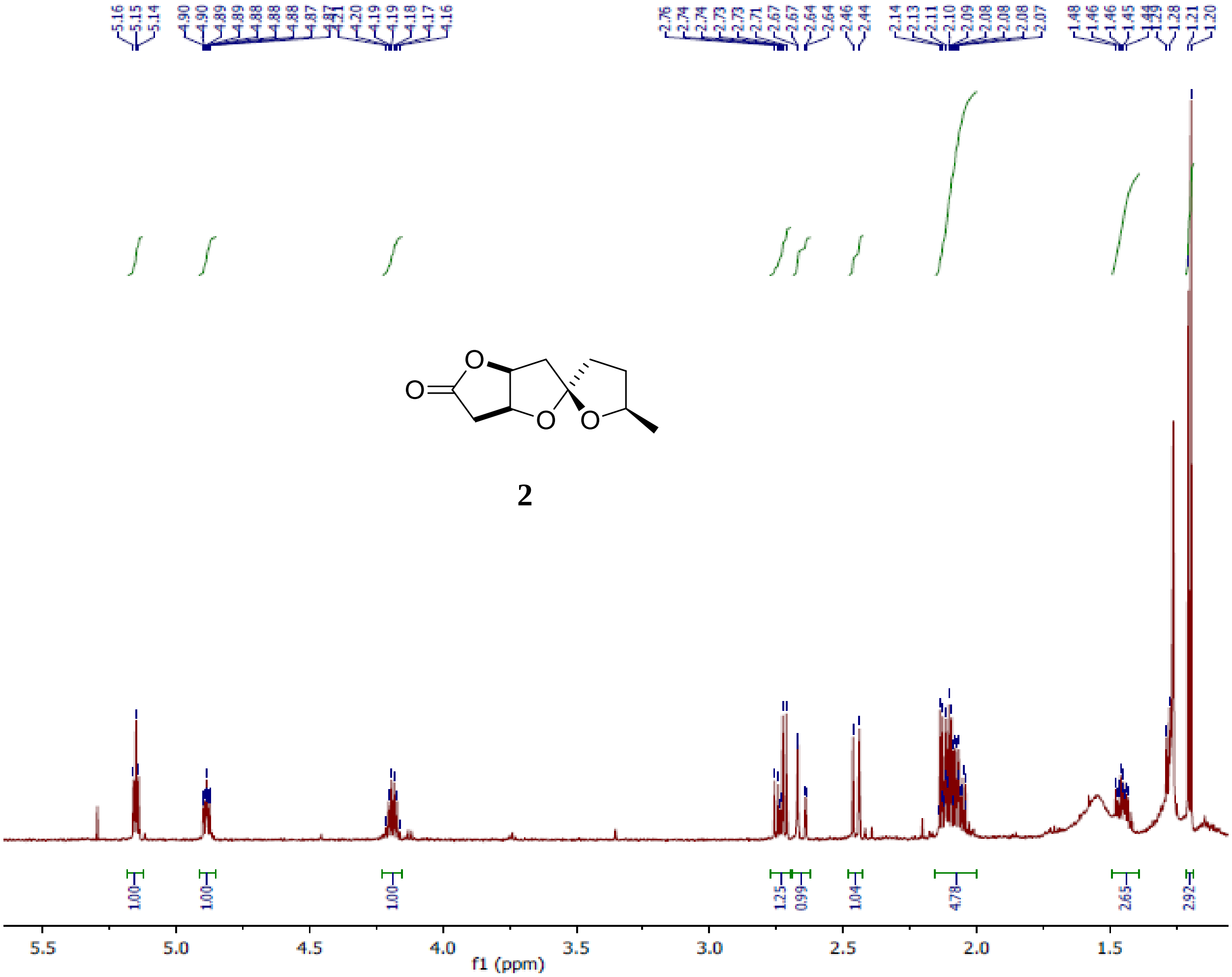

5.16 5.15 5.14
4.90 4.89 4.89 4.88 4.88 4.88 4.87
4.20 4.19 4.19 4.18 4.17 4.16
2.76 2.74 2.74 2.73 2.73 2.71 2.67 2.67 2.64 2.64 2.46 2.44
2.14 2.13 2.11 2.10 2.09 2.08 2.08 2.08 2.07
1.48 1.46 1.46 1.45 1.28 1.21 1.20
O
O
O
O
2
1.00
1.00
1.00
1.25
0.99
1.04
4.78
2.65
2.92
5.5 5.0 4.5 4.0 3.5 3.0 2.5 2.0 1.5
f1 (ppm)

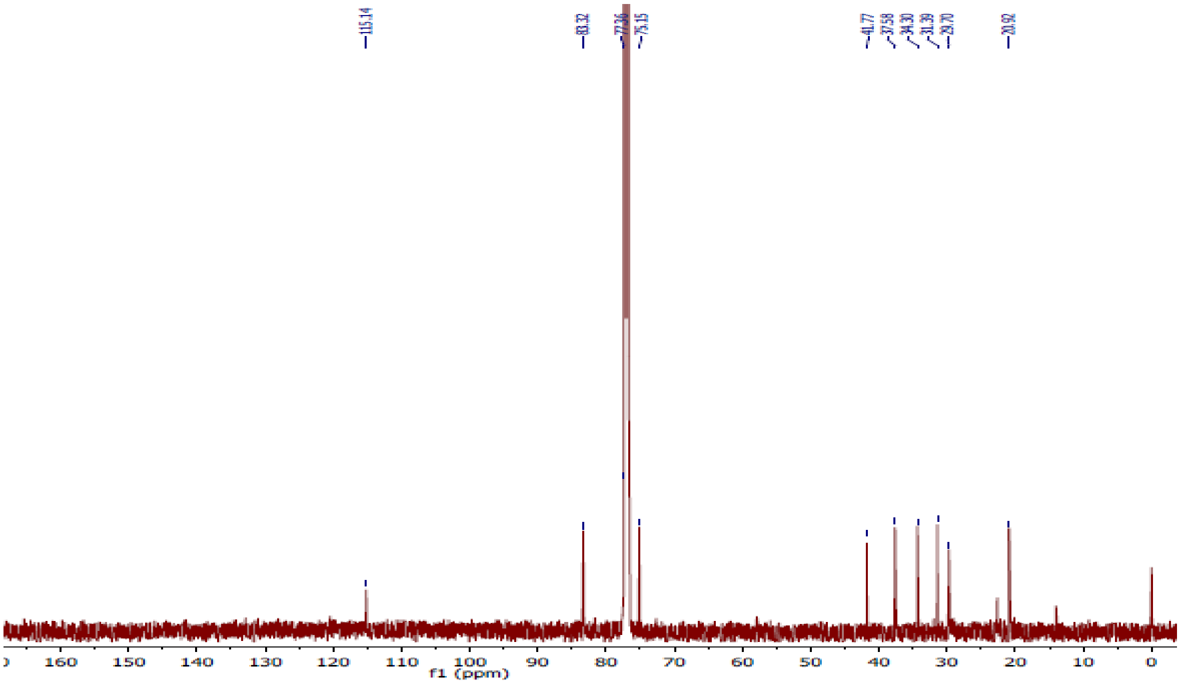

115.14
83.32
77.36
75.15
41.77 37.58 34.30 31.39 29.70
20.92
160 150 140 130 120 110 100 90 80 70 60 50 40 30 20 10 0
f1 (ppm)

References

1. Suntornchashwej, S.; Suwanborirux, K.; Isobe, M. *Tetrahedron* **2007,** *63*, 3217–3226.
